# Cardiometabolic risk factors in vegans; A meta-analysis of observational studies

**DOI:** 10.1371/journal.pone.0209086

**Published:** 2018-12-20

**Authors:** Jocelyne R. Benatar, Ralph A. H. Stewart

**Affiliations:** Green Lane Cardiovascular Service, Auckland City Hospital, Auckland, New Zealand; Tufts University, UNITED STATES

## Abstract

**Background:**

There is increasing evidence that plant based diets are associated with lower cardiovascular risk.

**Objective:**

To evaluate effects of a vegan compared to an omnivorous diet on cardio-metabolic risk factors.

**Methods:**

Meta-analysis of observational studies published between 1960 and June 2018 that reported one or more cardio-metabolic risk factors in vegans and controls eating an omnivorous diet were undertaken. Macro-nutrient intake and cardio-metabolic risk factors were compared by dietary pattern. The Newcastle Ottawa Scale (NOS) was used to assess the quality of each study. The inverse-variance method was used to pool mean differences. Statistical analyses were performed using RevMan software version 5•2 (The Nordic Cochrane Centre, The Cochrane Collaboration, Copenhagen.

**Results:**

40 studies with 12 619 vegans and 179 630 omnivores were included. From food frequency questionnaires in 28 studies, vegans compared to omnivores consumed less energy (-11%, 95% confidence interval -14 to -8) and less saturated fat (- 51%, CI -57 to -45). Compared to controls vegans had a lower body mass index (-1.72 kg/m^2^, CI -2.30 to -1.16), waist circumference (-2.35 cm, CI -3.93 to -0.76), low density lipoprotein cholesterol (-0.49 mmol/L CI -0.62 to -0.36), triglycerides (-0.14 mmol/L, CI -0.24 to -0.05), fasting blood glucose (-0.23 mmol/, CI -0.35 to -0.10), and systolic (-2.56 mmHg, CI -4.66 to -0.45) and diastolic blood pressure (-1.33 mmHg, CI -2.67 to -0.02), p<0.0001 for all. Results were consistent for studies with < and ≥ 50 vegans, and published before and after 2010. However in several large studies from Taiwan a vegan diet was not associated with favourable cardio-metabolic risk factors compared to the control diets.

**Conclusion:**

In most countries a vegan diet is associated with a more favourable cardio- metabolic profile compared to an omnivorous diet.

## Introduction

Dietary habits are an important determinant of health. According to current guidelines, a healthy dietary pattern is high in vegetables, fruit, whole grains, seafood, legumes, and nuts, and includes a modest amount of low- and non-fat dairy products. It is also low in red and processed meat, sugar-sweetened foods and beverages, and refined grains.[1,2] In the randomised Dietary Approach to Adults with systolic Hypertension (DASH) study, this dietary pattern was shown to reduce blood pressure,[3] and insulin resistance.[4] The Mediterranean diet is similar, but includes a higher intake of fruit and lower intake of dairy food. [5] The Mediterranean diet has been associated with reduced cardiovascular events,[6,7] diabetes,[8,9] obesity, lower blood pressure[9] and modest decrease in LDL cholesterol. [10] The ‘healthy vegetarian eating pattern’[1] has been associated with lower LDL- cholesterol [11,12] and blood pressure.[13] These diets include at least some dairy food, eggs and processed foods which may contain trans fatty acids and saturated fatty acids that affect lipid levels.[14] More recently the Prospective Urban Rural Epidemiological (PURE) study reported that a diet which included more dairy food and meat is associated with lower all cause and cardiovascular mortality in 138,000 people from lower, middle and high income countries.[15] This large study raises questions on whether and how dairy food and meat consumption may influence mortality risk, including their impact on known cardio-metabolic risk factors.

In contrast to most other dietary patterns, vegans generally strictly adhere to a plant based diet which avoids all animal products. Therefore, vegans provide an opportunity to assess the effects of a strict plant based diet on cardiometabolic risk factors. Cardiometabolic risk factors include increased waist circumference, low high density lipoprotein–cholesterol (HDL-c), high triglycerides, high blood pressure and insulin resistance. These risk factors are associated with increased risk of developing atherosclerotic cardiovascular (CV) disease and diabetes mellitus.[16] A literature review identified a number of studies which have reported these risk factors in vegans compared to omnivores, but most were small and evaluated only some risk factors. Also, studies have been undertaken over many years during which dietary patterns have often changed, and in diverse geographies. This meta-analysis was therefore undertaken to more reliably evaluate effects of a vegan diet on different cardiometabolic risk factors, and to determine whether associations are consistent across diverse populations, and over time.

## Methods

### Assessment of study eligibility and data extraction

The review was conducted according to Meta-analysis Of Observational Studies in Epidemiology (MOOSE) statement. [17] A protocol was developed and is available as a supplementary document. The search strings used are listed in the protocol (S2 File). Searches were performed of literature published from 1960 through to June 2018 using Medline, PubMed, Science Direct, Embase, Google, reference lists of articles, and proceedings of major meetings for relevant literature. The search terms were ‘vegan’ or ‘vegetarian’ and each of the following; ‘cardiometabolic risk’, ‘cardiovascular’, ‘weight’, ‘glucose’, ‘insulin’, ‘insulin resistance’, ‘blood pressure’, ‘cholesterol’ and ‘lipids’. It became apparent that some vegan studies were coded under the term ‘vegetarian’, so this was added to the search items.

A search was performed for all observational studies that reported any cardio-metabolic risk factor in healthy adults following a vegan diet longer than 6 months and also reported a control group who ate an omnivorous diet. The definition of ‘vegan’ varied between studies and was noted. Healthy adults were defined as those aged over 18 with no renal disease, diabetes and heart disease, or other significant comorbidities and who are not taking lipid, glucose or blood pressure lowering medication. There was no upper age restriction for participants for the metaanalysis.

Studies needed to include sufficient data to calculate estimates of effect with standard deviations on at least one of the following: body mass index, waist circumference, blood pressure, triglycerides, LDL cholesterol, fasting glucose and insulin resistance. We restricted inclusion to studies of healthy adults who did not have diabetes, hypertension or vascular disease and were not on lipid or glucose lowering medication. Studies were excluded if they included any other intervention or they were commentaries, reviews, were not in English, or were duplicate publications from the same study.

Both (JB, RS) reviewers screened abstracts, titles and when appropriate full text to determine eligibility. For eligible studies data were abstracted by JB in duplicate. Questions arising during data abstraction were resolved by discussion. Through an iterative process, a standard list was used to extract descriptive, methodological and key variables from all eligible studies.

Data extracted included year of publication, the primary aim of the study, population characteristics, funding source, age and gender, whether a food frequency questionnaire was used, how long patients were vegan, estimates of effect and standard deviations. If data was not included in the published report corresponding authors were contacted. [18–20] Studies that present results separately for males and females,[21–27] or pre and post-menopausal women[28] are treated as separate studies. The Newcastle Ottawa Scale (NOS) was used to assess the quality of each study[29]. Using this scale, each study is judged on eight items, categorized into three groups: the selection of the study groups; the comparability of the groups; and how diet pattern was ascertained (objectively or subjectively).Stars are awarded for each quality item and the highest quality studies are awarded up to nine stars. A study is considered of good quality if there are 3 or 4 stars in selection domain AND 1 or 2 stars in comparability domain AND 2 or 3 stars in outcome/exposure domain.

#### Statistical analysis

The inverse-variance method was used to pool mean differences to yield an overall effect size with 95% confidence intervals. For two studies where standard deviations or confidence intervals were not available despite contacting authors, (Fraser 2015 and Appleby),[12,19] the mean SD of all other studies was used.

For studies that present results of food frequency questionnaires, total energy (kilojoules), carbohydrate, total, saturated, polyunsaturated and mono unsaturated fat and protein intake (grams/day) were calculated. The mathematical weighted mean of each risk factor and for total energy, fat, protein and carbohydrate intake was calculated as follows = ∑(x×n)N, where x=meanofeachstudyN=totalnumberofparticipantsinmetanalysis and n=numberofparticipantsinthestudy.

Each meta-analysis was assessed for heterogeneity by a Chi square test and *I*^*2*^ statistic. A fixed effects model was used when heterogeneity was not present (*I*^*2*^ = 0) and a random effects model was used when statistical heterogeneity (I^2^≥1%) was present. The meta-analysis was also repeated using a fixed effects model to assess the effects of small studies on results.[30] A p-value of <0.05 was considered statistically significant. Studies are presented in Forrest plots in order of statistical power.

Sensitivity analysis excluded studies that deviated significantly from the standard error of the total study result, and studies where baseline values differed significantly from the overall average.

Stratified analyses were conducted by size of study (<50 or >50 vegan participants), geography (North America, Europe, Asia and other), and date of publication (< 2000, 2000–2010, >2010). Funnel plots were used to evaluate for possible publication bias.[31]However, Asian studies were found to be different across all measures so results are reported separately for Asian and non-Asian cohorts.

The Statistical analyses were performed using RevMan software version 5·2 (The Nordic Cochrane Centre, The Cochrane Collaboration, Copenhagen). Subgroup analysis followed guidelines suggested by Wang. [32]

The study was not funded.

## Results

### Summary of studies included

**Fig 1:**
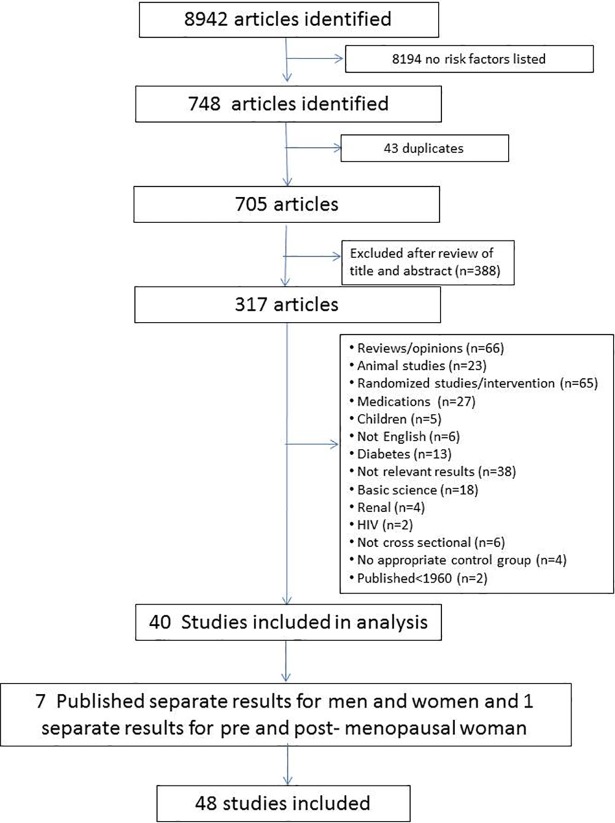
Study flow chart of meta-analysis of cross sectional studies.

**Fig 2:**
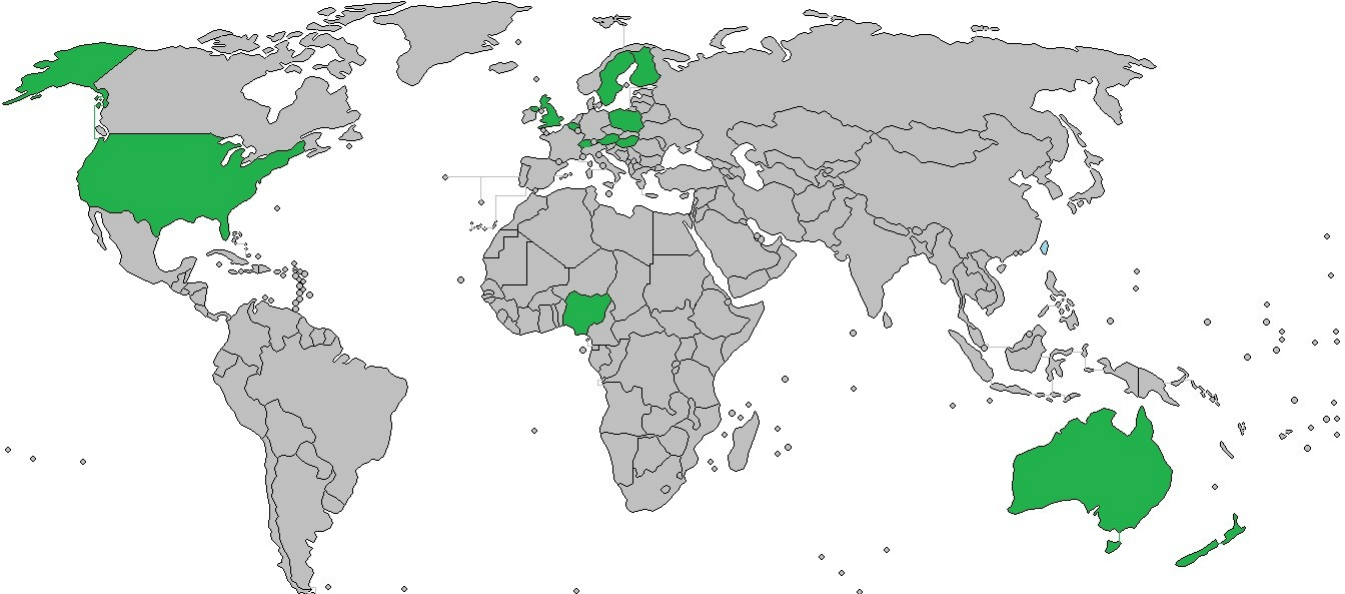
Countries that contributed to this meta-analysis.

The study flow chart is presented in Fig 1. Forty studies met inclusion criteria and were included in the meta-analysis with a total of 12 619 vegans and 179 630 omnivores (Table 1). Of these, 7 reported outcomes separately for male and females[21,23–27,33,34] and one for pre and post-menopausal woman, [28] that could not be combined, and are therefore treated as separate studies for a total of 48 studies. In all studies the vegan group had been on a vegan diet longer than 1 year, and all were funded publically except the study by Li et al.[35] The countries involved in the studies are listed in Table 1 and in Fig 2.

**Table 1:** Cross sectional studies included in the meta-analysis.

Trial Country Year published	Population	Vegans Number (% female)	Omnivores Number (% female)	Age in years Mean (SD)	Primary outcome	Risk factors assessed	Definition of vegan cohort	NOS Stars
Agren [36] Finland 1995	Adults Fins	8 (87%)	11(91%)	47 (12.1)	Lipids and phospholipids	BMI	Strictly uncooked vegan diet for many years with no animal products or added salt- ‘living diet’.	7
Appleby [12] United Kingdom 2002	EPIC– Oxford cohort.	739 (63%)	4737 (79%)	47 (12.1)	Blood pressure	BMI Systolic BP Diastolic BP	Identified via FFQ questions as not eating any meat, fish, eggs or dairy products	7
Benatar [37] New Zealand 2017	Health New Zealand adults	25 (76%)	61(74%)	33.5(8.1)	Cardiometabolic risk factors	BMI Waist circumference Fasting blood glucose Insulin resistance LDL-cholesterol Triglycerides Systolic BP Diastolic BP	Self-identified vegans recruited via vegan Facebook group. Confirmed with FFQ and fatty acid levels of 17:0 and 15:0.	8
Bradbury [38] United Kingdom 2014	EPIC– Oxford cohort.	422 (NR)	424(NR)	45 (12.0)	Lipids	BMI LDL-cholesterol	Identified via FFQ questions as not eating any meat, fish, eggs or dairy products	7
Chiu[39] Taiwan 2016	MJ Health Screening Centre	1913(75%)	40 915(75%)	48.9 (12.5)	Metabolic syndrome	BMI Waist circumference Fasting blood glucose LDL-cholesterol Triglycerides Systolic BP Diastolic BP	Identified via FFQ questions as not eating any meat, fish, eggs or dairy products	8
De Biase[40] Brazil 2005	Seventh Day Adventists in São Paulo	18 (45%)	22 (68%)	34.0 (13.0)	Lipids	BMI Waist circumference LDL-cholesterol Triglycerides	Self-identified vegans recruited via vegetarian restaurants at Seventh-day Adventist Churches, among Hare-Krishna members, and at spiritualistic centres.	4
Elorinne [41] Finland 2016	Healthy Finnish adults 18–50 years	22 (72%)	19 (58%)	34.0 (13.0)	Nutritional status	BMI	Self-identified vegans found via Finnish Vegan Association’s monthly newspaper and via an online discussion forum	8
Famodu[42] Nigeria 1998	Seventh-Day Adventist Seminary Institute of West Africa	8 (0%)	40 (0%)	47.8 (1.7)	Blood pressure and lipids	BMI Fasting blood glucose Systolic BP Diastolic BP	Seminary students and lecturers where cafeteria provide the majority of meals–*habitual vegans*	7
Fisher[43] United States America 1986	Seventh day adventists	10 (NR)	25 (56%)	37 (10)	Lipid and platelet levels	LDL-cholesterol Triglycerides	Identified via FFQ as consuming eggs and dairy products never or infrequently (less than once per week),	6
Fokkema Males [26] Netherlands 2000	Healthy Dutch males 20–60 years	8(0%)	9 (0%)	38.5 (3.4)	Polyunsaturated fatty acids	BMI	Self-identified vegans recruited by advertisement in the periodicals of the Dutch Vegan Association and the Groningen University	8
Fokkema Females [26] Netherlands 2000	Healthy Dutch females 20–60 years	4 (100%)	6 (100%)	34.2 (12.5)	Polyunsaturated fatty acids	BMI	Self-identified vegans recruited by advertisement in the periodicals of the Dutch Vegan Association and the Groningen University	8
Fontana[44] United States America 2007	Sedentary vegans and omnivores	21(55%)	21(55%)	53.1 (11)	Cardiometabolic risk factors	BMI Fasting blood glucose LDL-cholesterol Triglycerides Systolic BP Diastolic BP	Self-identified vegans recruited though The St. Louis Vegetarian Society and a Raw Food online magazine	9
Fraser[19] United States America 2015	Adventist Health Study-2 (AHS-2)- African American cohort	51 (NR)	366 (NR)	NR	Cardiometabolic risk factors	Waist circumference LDL-cholesterol Triglycerides Systolic BP Diastolic BP	Identified via FFQ within 2–3 years—Vegan defined as those, who eat no animal products	8
Goff[45] United Kingdom 2004	Caucasian subjects	21 (45%)	25 (50%)	42.4 (2.8)	Insulin and lipids	BMI Fasting blood glucose Insulin resistance LDL-cholesterol Triglycerides Systolic BP Diastolic BP	Self-identified vegan > 3 years recruited through an advertisement in The Vegan Society (UK) newsletter.	9
Gojda[46] Czechoslovakia 2013	Caucasian subjects	11 (45%)	10 (40%)	28.4 (3.2)	Insulin resistance	BMI Waist circumference Fasting blood glucose Insulin resistance LDL-cholesterol Triglycerides	Unclear how vegans identified for study. Vegans defined as no animal product > 3 years.	4
Haddad [47] United States America 1999	Seventh Day Adventist (University students)	25(60%)	20(50%)	34.8 (8.1)	Dietary and nutritional status	BMI LDL-cholesterol Triglycerides	Not clear- suggests vegan identified based on results of 4 day prospective FFQ.	5
Huang[18] Taiwan 2011	Elderly Nutrition and Health Survey in Taiwan (1999–2000)	83 (84%)	802 (40·5%)	71.9 (5.7)	Metabolic syndrome	BMI Fasting blood glucose LDL-cholesterol Systolic BP Diastolic BP	Identified via FFQ—three vegetarian meals/d and 30 d per month. 1 question asked what type of vegetarian diet. -h*abitual vegans*	8
Huang Pre menopausal[28] Taiwan 2014	Taiwanese Survey on Hypertension,Hyperglycemia, and Hyperlipid- emia (TwSHHH)	36 (100%)	2285 (100%)	41.1 (7.2)	Lipids	Waist circumference Fasting blood glucose LDL-cholesterol Triglycerides Systolic BP Diastolic BP	Identified via FFQ—diet excluded egg, milk, meat, poultry, seafood and by-products of animal slaughter for more than 1 year	8
Huang Postmenopausal[28] Taiwan 2014	Taiwanese Survey on Hypertension,Hyperglycemia, and Hyperlipid- emia (TwSHHH)	63 (100%)	1040 (100%)	62.2 (10)	Lipids	Waist circumference Fasting blood glucose LDL-cholesterol Triglycerides Systolic BP Diastolic BP	Identified via FFQ -excluded egg, milk, meat, poultry, seafood and by-products of animal slaughter for more than 1 year	8
Jian Females [33] Taiwan 2014	Taiwanese Survey on Hypertension,Hyperglycemia, and Hyperlipid- emia (TwSHHH)	99 (100%)	3325(100%)	50.2 (13.6)	Lipids and BP	Waist circumference Fasting blood glucose LDL-cholesterol Triglycerides Systolic BP Diastolic BP	Identified via FFQ -excluded egg, milk, meat, poultry, seafood and by-products of animal slaughter for more than 1 year	8
Jian Males [33] Taiwan 2014	Taiwanese Survey on Hypertension,Hyperglycemia, and Hyperlipid- emia (TwSHHH)	45 (0%)	3144(0%)	47.6 (16.3)	Lipids and BP	Waist circumference Fasting blood glucose LDL-cholesterol Triglycerides Systolic BP Diastolic BP	Identified via FFQ -excluded egg, milk, meat, poultry, seafood and by-products of animal slaughter for more than 1 year	8
Key Males[27] United Kingdom 2014	Oxford Vegetarian Study + EPIC-Oxford cohort	862 (0%)	8474 (0%)	43.8 (14)	Cancer rates	BMI	Identified via FFQ questions as not eating any meat, fish, eggs or dairy products–from GP practices and vegan society	7
Key Females[27] United Kingdom 2014	Oxford Vegetarian Study + EPIC-Oxford cohort	1384(100%)	24017(100%)	43.8 (14)	Cancer rates	BMI	Identified via FFQ questions as not eating any meat, fish, eggs or dairy products–from GP practices and vegan society	7
Krajcovicová-Kudlácková [48] Slovakia 2000	Healthy Slovakians	32 (69%)	59(63%)	41.2(5.8)	Homocysteine and lipids	BMI LDL-cholesterol Triglycerides	Slovak vegetarian society and boarders of the centre of healthy nutrition in Bratislava ‘who only ate plant food’	4
Kritchevsky[49] United States America 1984	Seventh-day Adventists	18 (50%)	25 (48%)	Not stated	Lipids	LDL-cholesterol Triglycerides	Identified via FFQ—eat no animal products	6
Kuchta [50] Poland 2016	Gdansk healthy adults	21 (57%)	21 (62%)	28 (5)	Lipids	BMI LDL-cholesterol Triglycerides Systolic BP Diastolic BP	Identified via FFQ—eat no animal products for at least 10 months- unclear how recruited	5
Li [35] Australia 1999	Males from Melbourne	18 (0%)	60 (0%)	34.5 (13.1)	Thrombotic risk factors	BMI LDL-cholesterol Triglycerides Systolic BP Diastolic BP	Self-identified vegans recruited from adverts; vegan was defined as someone who ate no meat and eggs and dairy products less than six times per year on FFQ.	8
Lin [51] Taiwan 2010	Buddhist nuns	102 (100%)	102 (100%)	46.6 (16.8)	Renal functions	BMI Fasting blood glucose LDL-cholesterol Triglycerides Systolic BP Diastolic BP	Vegetarian nuns- mainly only eat vegan food but occasionally consume dairy and eggs- *habitual vegans*	7
Newby[52] Sweden 2005	Swedish Mammography Cohort.	83 (100%)	54257 (100%)	53.5 (9.7)	Weight, BMI	BMI	Identified via FFQ zero consumption of meat, fish, eggs, and dairy products, respectively in large mammogram cohort	9
Orlich[53] United States America 2013	Adventist Health Study 2 (AHS-2)	5548 (64%)	35359 (66%)	56.3(13.6)	Mortality	BMI	Identified via FFQ—from churches in the United States and Canada between 2002 and 2007.	8
Orlov[54] Finland 1994	Finnish adults	9 (NR)	11(NR)	50(10)	Univalent cation fluxes in human erythrocytes	Systolic BP Diastolic BP	Strictly uncooked vegan diet for many years with no animal products or added salt- ‘living diet’	7
Pettersen[55] United States 2012	Adventist Health Study-2 (non-African American cohort)	49 (71.4%)	198 (61.6%)	62·7 (12.8)	Blood pressure	BMI Systolic BP Diastolic BP	Identified via FFQ—from churches in the United States and Canada between 2002 and 2007.	8
Roshanai Males [23] United kingdom 1984	NR	11(0%)	12(0%)	NR	Fatty acid intakes	LDL-cholesterol Triglycerides	Self-identified vegans from vegan society	6
Roshanai Females [23] United kingdom 1984	NR	12 (100%)	12 (100%)	NR	Fatty acid intakes	LDL-cholesterol Triglycerides	Self-identified vegans from vegan society	6
Sambol[56] Croatia 2009	Croatian adults	20	50	35.5(3.2)	Bone Density	BMI LDL-cholesterol Triglycerides	Not defined where vegans sourced.	4
Sanders[57] United Kingdom 1978	Healthy British adults	22 (45%)	22 (45%)	38 (12)	Phospholidids	BMI LDL-cholesterol Triglycerides	Self-identified vegans from vegan society	7
Sanders Males [24] United Kingdom 1987	Healthy British men	11 (5%)	11(0%)	30 (3.2)	Blood pressure, aldosterone, renin	BMI LDL-cholesterol Triglycerides	Self-identified vegans from vegan society	7
Sanders Females [24] United Kingdom 1987	Healthy British women	11 (100%)	11 (100%)	30 (3.2)	Blood pressure, aldosterone, renin	BMI LDL-cholesterol Triglycerides	Self-identified vegans from vegan society	7
Sanders Males [25] United Kingdom 1992	Healthy British men	10 (0%)	10(0%)	32	Platelet phospholipid fatty acid composition and function	BMI LDL-cholesterol Triglycerides	Self-identified vegans from vegan society	7
Sanders Females [25] United Kingdom 1992	Healthy British women	10(100%)	10(100%)	32	Platelet phospholipid fatty acid composition and function	BMI LDL-cholesterol Triglycerides	Self-identified vegans from vegan society	7
Schüpbach [58] Switzerland 2015	Healthy Swiss adults	53 (60%)	100(63%)	30.5(8.6)	Micronutrient status	BMI	Self-identified vegans using advertisements in schools, restaurants and shops.	6
Timko[59] United States America 2012	University students	35 (86%)	265 (70%)	24.89 (12.4)	Dietary restraint and eating disorder symptoms	BMI	Self-identified vegans from psychology department research pools of two urban universities, via flyers distributed to local health food stores, and through the internet	7
Thomas[60] United Kingdom 1999	European Prospective Investigation into Nutrition and Cancer	105 (100%)	153(100%)	47.8 (12.8)	Oestradiol and sex hormone-binding globulin	BMI	Self-identified vegans recruited through vegetarian and health food magazines, the Vegetarian Society and the Vegan Society, and word of mouth	8
Thorogood Males[21] United Kingdom 1990	Healthy British men	26(0%)	26 (0%)	42.5 (5.7)	Lipids	BMI LDL-cholesterol Triglycerides	Self-identified vegans recruited through the Vegetarian Society, through publicity in national and local media and word of mouth	8
Thorogood Females[21] United Kingdom 1990	Healthy British females	26(100%)	26 (100%)	42.5 (5.7)	Lipids	BMI LDL-cholesterol Triglycerides	Self-identified vegans recruited through the Vegetarian Society, through publicity in national and local media and word of mouth	8
Toohey Males[34] United States America 1998	African American Seventh-day Adventist	14 (0%)	49 (0%)	47.5 (12)	Cardiometabolic risk factors	BMI	Identified via FFQ -African American Church members from Washington, DC; Philadelphia, PA; and Baltimore, MD.	8
Toohey Females[34] United States America 1998	African American Seventh-day Adventist	31 (100%)	94 (100%)	51.5 (12)	Cardiometabolic risk factors	BMI Waist circumference	Identified via FFQ -African American Church members from Washington, DC; Philadelphia, PA; and Baltimore, MD.	8
Vinagre[20] Brazil 2013	Healthy adults	21(47%)	29 (41.3%)	36 (10)	Regulation metabolism of triglyceride rich lipoprotein	BMI LDL-cholesterol Triglycerides	Self-identified vegans from web site for vegetarians	6

Abbreviations: M = Male, F = female, pre-M = premenopausal, Post M = post-menopausal, BMI = body mass index, BP = blood pressure, NR = not reported

Most studies were of high quality as assessed by the Newcastle-Ottawa Scale (NOS) with a mean 7.1 Standard deviation (SD) 1.3 stars- the domain which consistently had the lowest star rating was for ascertainment of outcome. Most studies did not objectively measure diet and were dependent on self-reported intake. Few studies measured biomarkers such as fatty acids. A few studies scored low on the scale because it was not clear how the vegan or control population was sourced, so possible selection bias could not be assessed.

33 (69%) studies included less than 50 vegans (Table 2) and the majority of these were published before 2010. The three largest studies were the Adventist Health Study 2 (n = 5548 vegans), [53] the EPIC–Oxford studies (n = 739,422, and 2246 vegans respectively), [12,27,61] and the MJ Health database study (n = 1913 vegans). [39] All studies included an equivalent or greater number of controls compared to vegans. Eight studies reported separate outcomes for males (n = 987 vegans and 11 735 controls) and females (n = 1577 vegans and 27,498 controls) and are reported separately.[21–27,33,62] All studies were publically funded except Li et al [35] which was funded by the meat industry in Australia. Three studies required contact with authors for further data.[19,33,39]

**Table 2:** Summary of studies included in the meta-analysis.

Study Characteristics	Number of studies	Total number of vegans (% of all vegans)	Total number omnivores (% of all vegans)	Non-Asian			Asian		
				*studies*	*vegans*	*omnivores*	*studies*	*vegans*	*omnivores*
**Total Number of Studies**	48	12 619 (100%)	179 630 (100%)	41	10 134	129 062	7	2 485	50 568
**Studies <50 vegans**	34	671 (5%)	6 637 (4%)	32	635	1 208	2	81	5 429
**Studies > 50 vegans**	14	12 619 (95%)	179 630 (96%)	9	9 544	127 854	5	2 404	45 139
**Published <2010**	29	1 920 (15%)	60 238 (34%)	29	1 920	60 238	0	0	0
**Published ≥ 2010**	19	10 699 (85%)	119 392 (66%)	12	8 214	68 824	7	2 485	50 568
**Cardiometabolic Risk factor evaluated**									
Body mass index	37	12 241(97%)	169 385 (94%)	34	9 999	12 8611	3	2 242	40 774
Waist circumference	10	2 288 (18%)	50 571 (28%)	5	132	582	5	2 156	49 989
Fasting glucose	13	2 448 (19%)	51 798 (29%)	6	107	185	7	2 485	50 568
LDL- cholesterol	31	3 355 (27%)	53 393 (30%)	24	1 014	1 780	7	2 485	50 568
Triglycerides	29	2 731 (22%)	51 814 (29%)	23	473	1 003	6	2 258	50 811
Systolic and diastolic blood pressure	19	3 222 (26%)	53 870 (30%)	12	881	2 257	7	2 485	50 568

Two authors responded [19,33,39] and provided additional measurement such as lipids, weight and BP.

Asian studies [18,28,33,39] contributed the most participants for all cardiometabolic risk factors except BMI. These studies also contributed most participants for the sub group analysis based on studies published ≥ 2010 and those with > 50 vegans. All Asian studies were from Taiwan. The largest ones derive from two large databases; the Taiwanese Survey on Hypertension, Hyperglycemia, and Hyperlipidemia (TwSHHH)[18,28,33] and the MJ screening centre.[39]

### Macro-nutrient and energy intake

The definition of vegan for one Asian [33] and all non-Asian studies was avoidance of all animal flesh and by-product. For the other Asian studies,[18,28,35,39] the definition was less restrictive being defined as consumption of non-animal based food 3 times a day for 30 days a month. One study [20] reported results that were outside the normal range (for example total protein in each group was 15g per day which is 3 times less than for other studies). Authors for this study were contacted to address these disparities but they did not respond so it is not possible to account for these differences.

**Fig 3:**
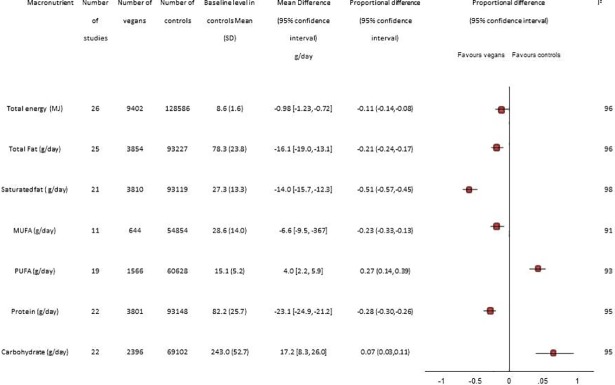
Macronutrient intake in vegans compared to omnivores.

Vegan status was supported by food frequency questionnaires in most studies (n = 26, 63%). The mean and proportional difference in intake of major macronutrients by dietary pattern is presented in Fig 3 and Figures A-G in S1 File. Proportional differences for individual studies are presented in Table 3 and subgroup analysis in Table 4. The mean daily energy intake was 11% less for than vegans compared to omnivores (8610KJ versus 7700KJ/day, respectively). Compared to omnivores vegans consumed less total fat (-16.06g, -18.98 to -13.13, p><0.0001), less saturated fat (-14.0g, CI -15.7 to -12.3), less mono-unsaturated fat (-6.6 g, CI -9.56 to -3.7) but more polyunsaturated fat (+4.0g, 2.2 to 5.9) (Table 3). Compared to omnivores, vegans also consumed less protein (-23.1g, CI -24.9 to -21.2) but more carbohydrate (+13.6g, CI 4.3 to 22.9) (p <0.0001 for all comparisons). (Table 3)

**Table 3:** Proportional intake of macronutrients in vegans compared to omnivores.

	Total energy	Total Fat	Saturated fat	MUFA	PUFA	Carbohydrate		Protein
Agren [36]	1.09	1.01	0.36	1.40	1.74	1.23		0.77
Appelby M [12]	0.88	0.91	0.47	NR	1.00	1.09		0.80
Appelby F [12]	0.89	0.91	0.52	NR	1.33	1.13		0.77
Bradbury M [61]	0.86	0.91	0.50	0.79	1.45	1.19		0.81
Bradbury F [61]	0.87	0.94	0.64	0.78	1.44	1.12		0.76
Famodu [42]	1.05	1.00	0.47	NR	1.27	1.05		0.75
Fontana[44]	1.17	1.12	0.71	1.47	1.37	0.87		0.54
Goff [45]	0.94	0.90	0.47	0.90	1.29	1.24		0.75
Gojda [46]	1.04	0.85	NR	NR	NR	1.21		0.82
Haddad M [47]	1.03	0.81	0.50	0.63	0.93	NR		NR
Haddad F [47]	0.86	0.74	0.50	0.74	1.31	NR		NR
Huang 2011[18]	0.83	0.97	0.73	NR	0.91	1.02		1.00
Key F [27]	0.87	0.87	0.57	NR	NR	1.17		0.78
Key M [27]	0.87	0.90	0.55	NR	NR	1.09		0.81
Krajcovicová-Kudlácková [48]	0.77	0.92	0.46	0.83	1.15	NR		NR
Kritchevsky M [49]	0.93	0.73	NR	NR	NR	1.40		0.61
Kritchevsky F[49]	0.87	0.68	NR	NR	NR	1.33		0.75
Kuchta [50]	1.06	0.95	NR	NR	NR	1.32		0.64
Li [35]	0.74	0.75	0.38	0.56	1.50	1.43		0.74
Newby [63]	0.83	0.75	0.69	0.60	0.77	1.23		0.76
Orlich [53]	1.01	NR	NR	NR	NR	NR		NR
Orlov [54]	1.05	101.17	0.35	1.40	1.66	1.14		0.78
Thorogood M [21]	1.01	0.88	0.47	NR	1.83	1.22		0.77
Thorogood F[21]	0.98	0.94	0.52	NR	1.57	1.18		0.79
Toohey M [34]	0.79	0.88	0.63	NR	1.13	0.99		0.83
Toohey F [34]	0.97	0.99	0.73	NR	0.75	1.00		0.90
Vinagre [20]	0.77	0.76	0.38	1.01	1.36	1.21		0.91
**Total weighted** **difference**	**0.89**	**0.75**	**0.49**	**0.91**	**1.31**	**1.04**	**0.73**	

**Table 4:** Intake of macronutrients in vegans compared to omnivores.

Study Characteristics	Number of studies	Total energy (MJ)		Total Fat (g/day)		Saturated fat (g/day)		Protein (g/day)		Carbohydrate (g/day)	
		*Mean*	*p*	*Mean (SD)*	*p*	*Mean (SD)*	*p*	*Mean (SD)*	*p*	*Mean (SD)*	*p*
< 50 vegans [20–22,34–36,42,45–50,54]	18	-1.04 [-1.59, -0.49]	<0.0001	-12.67 [-22.18, -3.17]	0.009	-12.63 [-13.06, -12.20]	<0.0001	-22.80 [-32.00, -13.59]	<0.0001	20.67 [-5.64, 46.98]	0.12
>50 vegans [12,18,27,53,61,63]	8	-0.93 [-1.25, -0.60]	<0.0001	-18.00 [-20.84, -15.17]	<0.0001	-14.05 [-14.27, -13.82]	<0.0001	-24.04 [-25.59, -22.49]	<0.0001	-1.69 [-8.39, 5.01]	0.22
North America[34,47,49,53]	8	-0.79 [-1.34, -0.24]	0.005	-11.60 [-24.96, 1.75]	0.09	-5.63 [-7.52, -3.75]	<0.0001	-18.71 [-31.84, -5.59]	0.0005	31.67 [-9.72, 73.06]	0.13
Europe [12,21,27,36,45,46,48,50,52,54,61]	14	-1.11 [-1.28, -0.95]	<0.0001	-14.31 [-16.34, -12.29]	<0.0001	-14.11 [-14.31, -13.91]	<0.0001	-24.61 [-25.78, -23.44]	<0.0001	10.28 [3.71, 16.85]]	0.002
Asia [18]	1	-1.18 [-1.81, -0.56]	0.0002	-39.68 [-44.06, -35.30]	<0.0001	-2.90 [-4.26, -1.54]	<0.0001	-10.30 [-13.63, -6.97]	<0.0001	12.96 [7.01, 18.91]	<0.0001
Other [20,35,42]	3	-1.86 [-3.79, 0.07]	0.06	-27.34 [-73.20, 18.52]	0.24	-11.83 [-13.59, -10.07]	<0.0001	-10.30 [-13.63, -6.97]	0.04	8.58 [-4.82, 21.97]	0.21
Published <2010 [12,20,21,34–36,42,45,47–49,52,54]	17	-1.00 [-1.32, -0.69]	<0.0001	-14.86 [-20.05, -9.68]	<0.0001	-12.59 [-13.02, -12.16]	<0.0001	-23.31 [-27.84, -18.78]	<0.0001	12.87 [-0.00, 25.75]	0.05
Published ≥2010 [18,27,46,50,53,61]	9	-0.99 [-1.50, -0.48]	<0.0001	-17.63 [-21.42, -13.83]	<0.0001	-14.07 [-14.29, -13.84]	<0.0001	-23.78 [-25.79, -21.78]	<0.0001	9.01 [-0.99, 19.01]	0.08
**OVERALL**	**26**	**-0.98 [-1.23, -0.72]**	**<0.0001**	**-16.06 [-18.98, -13.13]**	**<0.0001**	**-13.99 [-15.67, -12.31]**	**<0.0001**	**-23.08 [-24.93, -21.22]**	**<0.0001**	**11.22 [4.11, 18.33]**	**0.004**

Total energy intake in vegans was 30% from fat (5.8% saturated), 13% protein and 56% carbohydrate. In controls 33% of total energy was from total fat, 11% saturated fat, 17% protein and 51% from carbohydrate. The nutrient intake was similar across studies including the sole study from Asia (Taiwan) that reported results of FFQ.[18] Differences in energy intake between vegans and controls were similar by publication date, geography or size of study (Table 4).

### Cardiometabolic risk factors

**Fig 4:**
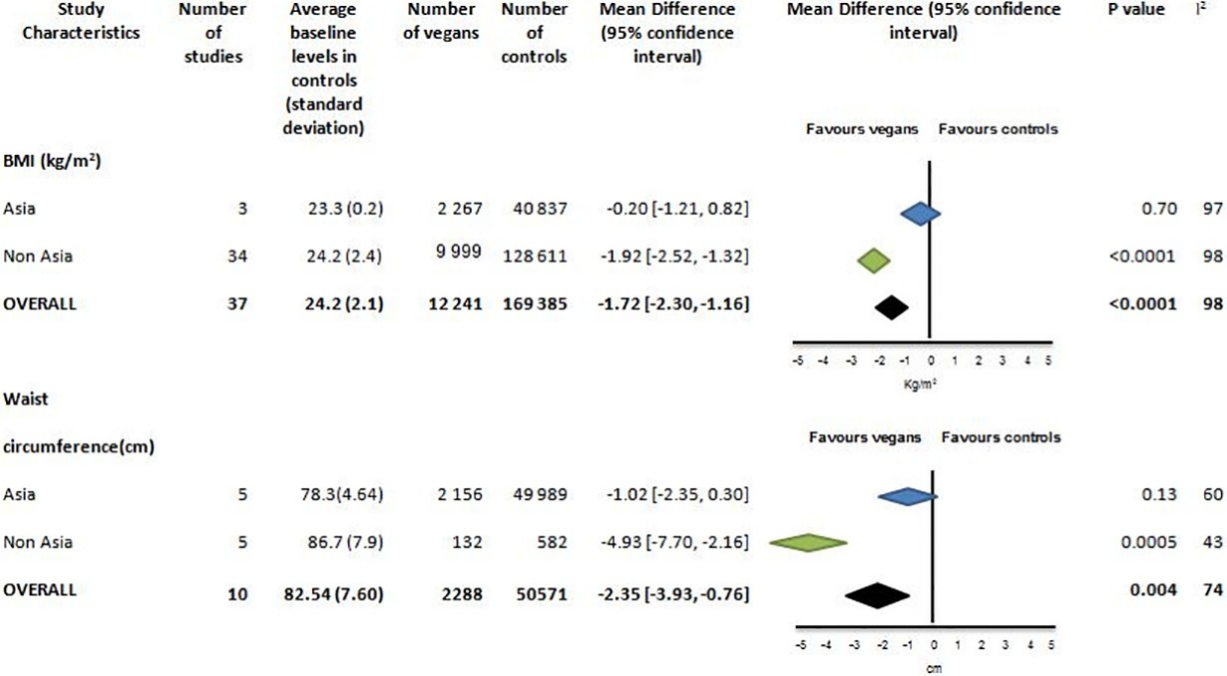
Body mass index (kg/m2) and waist circumference (cm) in vegans compared to omnivores.

**Fig 5:**
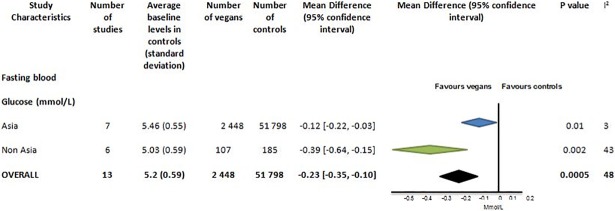
Fasting blood glucose (mmol/L) in vegans compared to omnivores.

**Fig 6:**
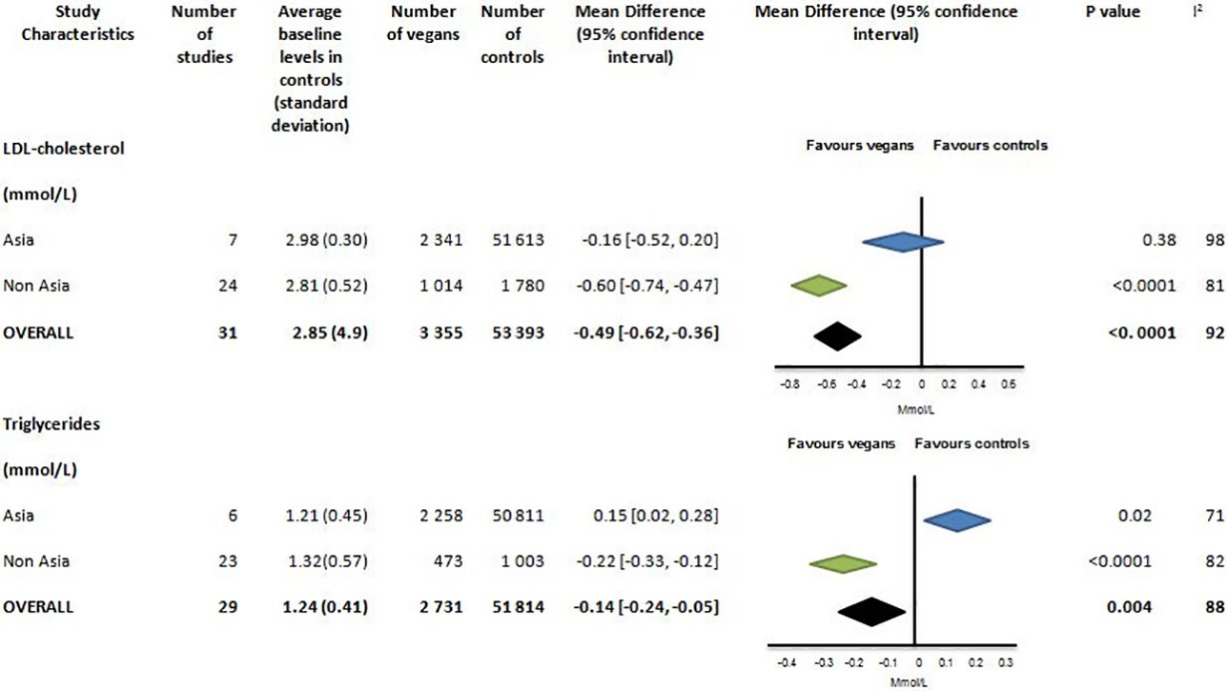
Low density lipoprotein cholesterol and triglycerides (mmol/L) in vegans compared to omnivores.

On pre-specified subgroup analysis based on geographic region, there was a statistically significant differences comparing Asian and non-Asian studies for all factors except blood glucose and diastolic blood pressure. For geographic regions excluding Asia there was no difference by year of publication or size of study. Results are therefore reported separately for Asian and non-Asian studies (Figs 3–6). Generally, for all risk factors Asian studies reported smaller or no difference in cardiometabolic risk factors between vegans and omnivores.

#### Body mass index

BMI was reported in 37 studies with 12 241 vegans and 169 385 controls (Fig 4 and Fig H in S1 File). The BMI of controls was within the healthy weight range, 24.2 ±1.2kg/m^2^. There was no difference in BMI for vegans compared to controls in Asia (-0.20 95% CI-1.21 to 0.82, p = 0.70) kg/m^2^, p = 0.92), but for non-Asian studies the difference was -1.92 kg/m^2^ (95% CI -2.52 to -1.32, p< 0.0001). There was significant heterogeneity in results (I^2^ = 98%) across these subgroups. However there is no suggestion of publication bias form funnel plots (Fig I in S1 File).

#### Waist circumference

Waist circumference was reported in 10 studies with 2 288 vegans and 50 571 controls (Fig 4 and Fig J in S1 File). Weighted mean waist circumference was 77.5 cm for omnivores and 76.2cm for vegans. There was no difference in waist circumference between vegans and omnivores in studies from Asian. For non-Asian studies waist circumference in vegans was -4.93 cm [-7.70 to -2.16] less than controls, p = 0.0005. However, there were only 132 vegans in the non-Asian cohort and 2 156 vegans in the Asian cohort. There was significant heterogeneity in the difference in waist circumference between vegans and controls between studies (I^2^ = 48%), but no evidence to suggest publication bias on the funnel plot (Fig K in S1 File).

#### Blood glucose and insulin resistance

Fasting blood glucose was reported in 13 studies with 2 448 vegans and 51 798 controls (Fig 5 and Fig LI in S1 File). Studies from Asia predominated with 94% of the total vegan population. The mean fasting plasma glucose in controls was 5.2 (0.59) mmol/L. The difference in fasting glucose overall was -0.23 [95% CI -0.35 to -0.10] mmol/l, p = 0.0005. Vegans in both Asian and non-Asian studies had reduced blood sugars and there was no statistical difference between these subgroups (p = 0.10). There was moderate heterogeneity between studies (I^2^ = 48%) and some suggestion of publication bias (Fig M in S1 File). Results are unchanged when the study by Vinagre [20] which reports the largest effect on fasting plasma glucose was excluded.

The Homeostatic model assessment—insulin resistance (HOMA-IR) was reported in 3 non-Asian studies with 57 vegans and 92 controls (Fig N in S1 File). The difference in HOMA-IR was -0.04 [95% CI -0.36 to 0.28] using the fixed effects model and there was no heterogeneity between studies (Fig O in S1 File).

#### LDL- cholesterol

LDL- cholesterol was reported in 31 studies with 3 355 vegans and 53 393 controls ((Fig 6 and Fig P in S1 File). For all studies the difference in LDL-cholesterol between vegans and controls was -0.49mmol/L [95% CI -0.62 to -0.36], p<0.0001.The mean LDL- cholesterol was 2.85 (4.9) mmol/L for controls. Asian controls had higher baseline LDL- cholesterol compared to non-Asians but this difference was not statistically significant (p = 0.32). There was no difference between vegans and controls in LDL-cholesterol in Asian studies. However, for non-Asian studies this was -0.60mmol/L [95% CI -0.74 to -0.47], p <0.0001. There was significant heterogeneity between results (I^2^ = 92%) (Fig Q in S1 File). Results were similar when the study with the greatest difference (> 1.2mmol/l) [25] was excluded (-0.48 [95% CI -0.61, -0.35]).

#### Triglycerides

Triglycerides were reported in 29 studies with 2 731 vegans and 51 814 controls ((Fig 6 and Fig R in S1 File). The mean triglyceride was 1.24 (0.41) mmol/L for omnivores. Vegans had lower triglyceride levels than controls -0.14 mmol/l [95% CI-0.24 to -0.05], p = 0.004. However in studies from Asia, the converse was true with vegans having higher triglycerides than controls (0.15 [95% CI0.02 to 0.28]; p = 0.02). Asian studies contributed 82% of all vegans in the analysis. There was significant heterogeneity between results (I^2^ = 88%) and some risk of publication bias from funnel plots (Fig S in S1 File).

#### Blood pressure

**Fig 7:**
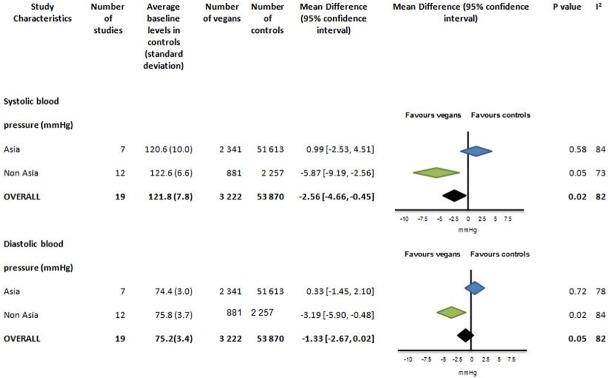
Systolic and diastolic blood pressure (mmHg) in vegans compared to omnivores.

Blood pressure was reported in 19 studies with 3 222 vegans and 53 870 controls (Fig 7, Figures T-W in S1 File). The mean systolic and diastolic blood pressure for controls was 121.8 (7.8) and 75.2(3.4) mmHg respectively. There was no difference in blood pressure between vegans and controls in Asian studies, which contributed 82% of the total vegan cohort with blood pressure data. In non-Asians studies systolic (-5.87mmHg [95% CI -9.19 to -2.56], p = 0.005) and diastolic blood pressure (-3.19mmHg [-5.90 to -0.48], p = 0.002) were lower in vegans compared to controls.

There was significant heterogeneity between results for both diastolic and systolic blood pressure (I^2^ = 82%). No effect on systolic and diastolic BP was seen when two non-Asian studies[44,54] with effects > 15mmHg were excluded from the analysis (mean difference -1.14 [95% CI -3.03, 0.75) mmHg).

#### Subgroup and sensitivity analysis

In pre-specified subgroup analysis there was no difference in results based on publication date or size of study once the impact of studies from Asia was taken into account. There was no overlap in confidence interval between Asia and Non-Asian studies for all risk factors except for fasting blood glucose and diastolic blood pressure. Results were similar for all sensitivity analysis performed where differences in risk factors differed significantly from the mean values of the whole population, and where studies reported outcomes much larger than the mean change. Results were unchanged within the Asian studies when subgroup analysis was done in restrictive compared to less restrictive definition of vegan.

## Discussion

In most countries vegans consumed less energy, total fat, saturated fat and protein compared to controls that ate an omnivorous diet. Vegans had a lower body mass index, LDL-cholesterol, blood glucose, triglycerides and blood pressure compared to healthy controls. However in studies from Taiwan, the only Asian country included in the meta-analysis, there was no difference in risk factors in vegans compared to controls with the exception of fasting blood glucose, which was slightly lower in vegans and triglycerides that were higher in vegans. These studies were large, contemporary and contributed substantially to the overall estimates, but results for ‘Asian’ and non-Asian studies differed significantly. It is therefore important to consider reasons for this geographic difference, and whether excluding studies from Taiwan provide more reliable estimates of the impact of a vegan diet in other countries.

The lack of difference in risk factors between vegans and omnivores in studies from Taiwan may reflect differences in the diet of vegans and/or controls compared to other populations.[64] Vegans included in the studies from Taiwan may adhere less strictly to a vegan diet. In these studies [18,28,35,39] the definition of vegan was less restrictive and was defined as consumption of non-animal based food 3 times a day, 30 days a month. When a subgroup analysis was done in restrictive compared to less restrictive definition of vegan in Asia, there was no difference in the result. This suggests that other factors may explain difference between Asian and non-Asian studies. For example, the diet pattern for omnivores in Asia may include less animal product than for non-Asian countries, so the differences between omnivores and vegans may be less. Diets across Asia are diverse, but there were no studies from other Asian countries which met inclusion criteria. Based on these observations the subgroup analysis which excludes Asian studies may provide a more reliable estimate of effects of a strictly vegan diet compared to a omnivorous diet in non-Asian countries.

The risk factor with the most evidence is body mass index, and this was consistently lower in vegans compared to controls in diverse geographies outside Asia, in larger and smaller studies, and in studies published over many years. Vegans consumed 980 less kilojoules per day which translated into 11% less energy than controls. Reduced energy intake rather than specific components of diet is the likely major reason for lower BMI and waist circumference in vegans.[65,66]

In non-Asian studies LDL cholesterol was 0.6mmol/l lower in vegans compared to controls based on observations in 1014 vegans from 24 studies. This reduction is consistent with effects of reducing saturated fat intake by 51%, and a 26% increase in PUFA intake compared to controls. [67,68] Avoidance of dairy food and meat [69] which may contain trans fatty acids, may also have a favourable effect on LDL cholesterol. In this study, it was not possible to assess the intake of processed foods that contain trans fatty acids in vegans or controls. Based on randomised trials of statins, a 0.6mmol/L reduction in LDL-cholesterol would be expected to reduce cardiovascular risk by ~19%. [70]

In non-Asian studies systolic blood pressure was ~ 6mmHg lower in vegans compared to controls. This would be expected to reduce cardiovascular risk by ~12%. [71] This lower blood pressure is similar to that observed in a meta-analysis of vegetarian diets.[72] It is possible lower body mass index is the principal reason for lower blood pressure, triglycerides and glucose for vegans compared to controls. [73–75] We were not able to determine whether features of the vegan eating patterns, [41,76] such as higher fruit and vegetable intake, [3] or low intake of processed foods high in salt [3] contributed to lower blood pressure in vegans.

In non-Asian studies, vegans had -0.39mmol/l lower fasting blood glucose compared to controls. This is also consistent with randomised studies that suggest that the vegan diet improves glycaemic control in people with type 2 diabetes.[77,78] It was not possible to confidently assess the association between the vegan diet and insulin resistance as too few studies have reported this.

For many studies, self-identified vegans were sourced from vegan societies and websites, with omnivores from the broader community. For others, both vegans and omnivores were sourced from religious institutions. This may have impacted on cardiometabolic risk factors in both vegans and omnivores. For example, vegans who belong to vegan societies are more likely to be strictly vegan and to consider themselves as ethical and political vegans. [79] These vegans also are more likely to engage in other health behaviours like exercising more and smoking less which may impact on cardiometabolic risk factors. The difference between vegans and omnivores may therefore be more marked. In contrast where study populations are sourced from religious institutes, the difference between vegans and omnivores in diet and health behaviours may be less. For example, Buddhist nuns are often habitual vegans rather than strictly vegan and their omnivorous counterparts eat a diet that is low in animal products. This may dilute differences between the groups. Differences between Asian vegans and omnivores may also be reduced as the Asian diet is traditionally low in animal products and high in carbohydrates.

A vegan diet has favourable effects on multiple risk factors, which would be expected to reduce CV risk much more than an intervention which influenced only one risk factor. However the size of the CV risk reduction is difficult to quantify.[80] Also it is possible the vegan diet has other effects on health and CV risk by mechanisms such as inflammatory pathways which were not assessed in this meta-analysis. Deficiencies in some nutrients such as vitamin B12, creatine, carnosine, taurine, vitamin D3, heme-iron and the omega-3 fatty acids may also influence cardiovascular health.[76]

### Limitations

It is possible some associations could be influenced by factors other than diet. Vegans may choose this diet because of perceived health benefits or religious/cultural reasons, and they may have fewer adverse health behaviours including smoking, drinking alcohol and sedentary lifestyle.[81]

Individual participant data was not available and this limits the ability to address a number of questions. We could not reliably evaluate possible gender differences, or associations between energy or saturated fat intake and cardiometabolic risk factors. Food frequency questionnaires are known to be unreliable, [82–84] and we could not assess the quality of the food consumed, including intake of processed food, trans fat and refined sugars. Most included studies were small, but results were similar in smaller and larger studies and by year of publication. Associations with triglyceride need to be interpreted with caution due to the significant scattered distribution seen in the funnel plot.

Larger cohort studies which evaluate a broad range of risk factors would overcome the limitation of small numbers, and additional studies in diverse populations would provide further information on the effects of a vegan diet compared to other diets. However even large observational studies will be limited by the potential for bias related to the impact of non-diet related factors. Randomised clinical trials which compare introduction of a vegan with omnivorous diet would provide more reliable information on effects on cardio-metabolic risk factors, but have not been undertaken. A limitation is that maintaining a vegan diet for a prolonged time as part of a clinical trial may be difficult for many people.

## Conclusion

In most countries a vegan diet has less energy and saturated fat compared to omnivorous control diets, and is associated with favourable cardiometabolic risk profile including lower body weight, LDL cholesterol, fasting blood glucose, blood pressure and triglycerides. These observations support other evidence that plant based diets are likely to lower the risk of cardiovascular disease and diabetes. However the improvement in cardiometabolic risk profile is also likely to depend on the comparison diet, and the difference may be less with some Asian compared to western dietary patterns.

## Supporting information

S1 File Supplementary document containing supplementary figures **Figure A**: Total energy intake (Mega joules) in vegans compared to omnivores **Figure B**: Total fat intake in grams per day in vegans compared to omnivores **Figure C**: Saturated fat intake in grams per day in vegans compared to omnivores **Figure D**: Monounsaturated fat intake in grams per day in vegans compared to omnivores **Figure E**: Polyunsaturated fat intake in grams per day in vegans compared to omnivores **Figure F**: Protein intake in grams per day in vegans compared to omnivores **Figure G**: Carbohydrate intake in grams per day in vegans compared to omnivores **Figure H**: Body mass index (kg/m2) in vegans compared to omnivores. **Figure I**: Funnel plot of body mass index (kg/m2) in vegans compared to omnivores. **Figure J**: Waist circumference (cm) in vegans compared to omnivores. **Figure K**: Funnel plot waist circumference (cm) in vegans compared to omnivores **Figure L**: Fasting blood glucose (mmol/L) in vegans compared to omnivores **Figure M**: Funnel plot fasting blood glucose (mmol/L) in vegans compared to omnivores **Figure N**: Insulin resistance as calculated by the homeostasis model (insulin resistance) in vegans compared to omnivores. **Figure O**: Insulin resistance as calculated by the homeostasis model (insulin resistance) in vegans compared to omnivores. **Figure P**: Low density lipoprotein cholesterol (mmol/L) level in vegans compared to omnivores. **Figure Q**: Funnel plot low density lipoprotein cholesterol (mmol/L) level in vegans compared to omnivores. **Figure R**: Triglyceride (mmol/L) levels in vegans compared to omnivores. **Figure S**: Funnel plot triglyceride (mmol/L) levels in vegans compared to omnivores. **Figure T**: Systolic blood pressure (mmHg) in vegans compared to omnivores. **Figure U**: Funnel plot systolic blood pressure (mmHg) in vegans compared to omnivores. **Figure V**: Diastolic blood pressure (mmHg) in vegans compared to omnivores. **Figure W**: Funnel plot diastolic blood pressure (mmHg) in vegans compared to omnivores.
(DOCX)

S2 File Study protocol. (DOCX)

S3 File Prisma 2009 checklist. (DOCX)

S4 File Newcastle—Ottawa Quality Assessment Scale (NOS Scale). (DOCX)

S5 File MOOSE checklist final. (DOCX)

## References

[1] (2015) 2015–2020 Dietary Guidelines for Americans. United States of America: USDA Center for Nutrition Policy and Promotion.

[2] Perk J, De Backer G, Gohlke H, Graham I, Reiner Z, et al (2012) European Guidelines on cardiovascular disease prevention in clinical practice (version 2012). The Fifth Joint Task Force of the European Society of Cardiology and Other Societies on Cardiovascular Disease Prevention in Clinical Practice (constituted by representatives of nine societies and by invited experts). Developed with the special contribution of the European Association for Cardiovascular Prevention & Rehabilitation (EACPR). Eur Heart J 33: 1635–1701. 10.1093/eurheartj/ehs092 22555213

[3] Appel LJ, Moore TJ, Obarzanek E, Vollmer WM, Svetkey LP, et al (1997) A clinical trial of the effects of dietary patterns on blood pressure. DASH Collaborative Research Group. N Engl J Med 336: 1117–1124. 10.1056/NEJM199704173361601 9099655

[4] Liese AD, Nichols M, Sun X, D'Agostino RB Jr., Haffner SM (2009) Adherence to the DASH Diet is inversely associated with incidence of type 2 diabetes: the insulin resistance atherosclerosis study. Diabetes Care 32: 1434–1436. 10.2337/dc09-0228 19487638 PMC2713612

[5] Estruch R, Ros E, Salas-Salvadó J, Covas MI, Corella D, et al (2013) Primary prevention of cardiovascular disease with a Mediterranean diet. N Engl J Med 368. 10.1056/NEJMc1806491 29897867

[6] Estruch R, Ros E, Salas-Salvado J, Covas MI, Pharm D, et al (2013) Primary Prevention of Cardiovascular Disease with a Mediterranean Diet. N Engl J Med: 1279–1290. 10.1056/NEJMoa1200303 23432189

[7] Kris-Etherton P, Eckel RH, Howard BV, St. Jeor S, Bazzarre TL, et al (2001) Lyon Diet Heart Study: Benefits of a Mediterranean-Style, National Cholesterol Education Program/American Heart Association Step I Dietary Pattern on Cardiovascular Disease. Circulation 103: 1823–1825. 11282918 10.1161/01.cir.103.13.1823

[8] Esposito K, Giugliano D (2014) Mediterranean diet and type 2 diabetes. Diabetes Metab Res Rev 30 Suppl 1: 34–40. 24357346 10.1002/dmrr.2516

[9] Pérez-Martínez P, García-Ríos A, Delgado-Lista J, Pérez-Jiménez F, López-Miranda J (2011) Mediterranean diet rich in olive oil and obesity, metabolic syndrome and diabetes mellitus. Curr Pharm Des 17. 10.2174/138161211795428948 21443484

[10] Rosenthal RL (2000) Effectiveness of altering serum cholesterol levels without drugs. Proceedings (Baylor University Medical Center) 13: 351–355. 16389340 10.1080/08998280.2000.11927704 PMC1312230

[11] Mann N, Pirotta Y, O'Connell S, Li D, Kelly F, et al (2006) Fatty acid composition of habitual omnivore and vegetarian diets. Lipids 41: 637–646. 17069347 10.1007/s11745-006-5014-9

[12] Appleby PN, Davey GK, Key TJ (2002) Hypertension and blood pressure among meat eaters, fish eaters, vegetarians and vegans in EPIC–Oxford. Public Health Nutrition 5: 645–654. 10.1079/PHN2002332 12372158

[13] Yokoyama Y, Nishimura K, Barnard ND, et al (2014) Vegetarian diets and blood pressure: A meta-analysis. JAMA Internal Medicine 174: 577–587. 10.1001/jamainternmed.2013.14547 24566947

[14] Hammad S, Pu S, Jones PJ (2016) Current Evidence Supporting the Link Between Dietary Fatty Acids and Cardiovascular Disease. Lipids 51: 507–517. 10.1007/s11745-015-4113-x 26719191

[15] Dehghan M, Mente A, Rangarajan S, Sheridan P, Mohan V, et al Association of dairy intake with cardiovascular disease and mortality in 21 countries from five continents (PURE): a prospective cohort study. The Lancet. 10.1016/S0140-6736(18)31812-9 30217460

[16] Cannon CP (2007) Cardiovascular disease and modifiable cardiometabolic risk factors. Clin Cornerstone 8: 11–28. 18452839 10.1016/s1098-3597(07)80025-1

[17] Stroup DF, Berlin JA, Morton SC, Olkin I, Williamson GD, et al (2000) Meta-analysis of observational studies in epidemiology: a proposal for reporting. Meta-analysis Of Observational Studies in Epidemiology (MOOSE) group. Jama 283: 2008–2012. 10789670 10.1001/jama.283.15.2008

[18] Huang CJ, Fan YC, Liu JF, Tsai PS (2011) Characteristics and nutrient intake of Taiwanese elderly vegetarians: evidence from a national survey. Br J Nutr 106: 451–460. 10.1017/S0007114511000195 21385505

[19] Fraser G, Katuli S, Anousheh R, Knutsen S, Herring P, et al (2015) Vegetarian diets and cardiovascular risk factors in black members of the Adventist Health Study-2. Public Health Nutr 18: 537–545. 10.1017/S1368980014000263 24636393 PMC4167463

[20] Vinagre JC, Vinagre CG, Pozzi FS, Slywitch E, Maranhao RC (2013) Metabolism of triglyceride-rich lipoproteins and transfer of lipids to high-density lipoproteins (HDL) in vegan and omnivore subjects. Nutr Metab Cardiovasc Dis 23: 61–67. 10.1016/j.numecd.2011.02.011 21937206

[21] Thorogood M, Roe L, McPherson K, Mann J (1990) Dietary intake and plasma lipid levels: lessons from a study of the diet of health conscious groups. Bmj 300: 1297–1301. 2369659 10.1136/bmj.300.6735.1297 PMC1663050

[22] Hardinge MGS, Fredrick J. (1954) Nutritional studies of vegetarians: dietary and serum levels of cholesterol The American Journal of Clinical Nutrition 2: 83–88. 13163186

[23] Roshanai F ST (1984) Assessment of fatty acid intakes in vegans and omnivores. Hum Nutr Appl Nutr 38: 345–354. 6526681

[24] Sanders TA, Key TJ (1987) Blood pressure, plasma renin activity and aldosterone concentrations in vegans and omnivore controls. Hum Nutr Appl Nutr 41: 204–211. 3305428

[25] Sanders TA, Roshanai F (1992) Platelet phospholipid fatty acid composition and function in vegans compared with age- and sex-matched omnivore controls. Eur J Clin Nutr 46: 823–831. 1425536

[26] Fokkema MR, Brouwer DA, Hasperhoven MB, Hettema Y, Bemelmans WJ, et al (2000) Polyunsaturated fatty acid status of Dutch vegans and omnivores. Prostaglandins Leukot Essent Fatty Acids 63: 279–285. 10.1054/plef.2000.0215 11090254

[27] Key TJ, Appleby PN, Crowe FL, Bradbury KE, Schmidt JA, et al (2014) Cancer in British vegetarians: updated analyses of 4998 incident cancers in a cohort of 32,491 meat eaters, 8612 fish eaters, 18,298 vegetarians, and 2246 vegans. Am J Clin Nutr 100 Suppl 1: 378s–385s. 24898235 10.3945/ajcn.113.071266 PMC4144109

[28] Huang YW, Jian ZH, Chang HC, Nfor ON, Ko PC, et al (2014) Vegan diet and blood lipid profiles: a cross-sectional study of pre and postmenopausal women. BMC Womens Health 14: 55 10.1186/1472-6874-14-55 24712525 PMC3996202

[29] Wells GA SB, O’Connell D, Peterson J, Welch V, Losos M, et al. The Newcastle-Ottawa Scale (NOS) for assessing the quality if nonrandomized studies in meta-analyses.

[30] Tufanaru C, Munn Z, Stephenson M, Aromataris E (2015) Fixed or random effects meta-analysis? Common methodological issues in systematic reviews of effectiveness. Int J Evid Based Healthc 13: 196–207. 10.1097/XEB.0000000000000065 26355603

[31] Egger M, Davey Smith G, Schneider M, Minder C (1997) Bias in meta-analysis detected by a simple, graphical test. Bmj 315: 629–634. 9310563 10.1136/bmj.315.7109.629 PMC2127453

[32] Wang R, Lagakos SW, Ware JH, Hunter DJ, Drazen JM (2007) Statistics in Medicine—Reporting of Subgroup Analyses in Clinical Trials. New England Journal of Medicine 357: 2189–2194. 10.1056/NEJMsr077003 18032770

[33] Jian ZH, Chiang YC, Lung CC, Ho CC, Ko PC, et al (2015) Vegetarian diet and cholesterol and TAG levels by gender. Public Health Nutr 18: 721–726. 10.1017/S1368980014000883 24963684 PMC10271072

[34] Toohey ML, Harris MA, DeWitt W, Foster G, Schmidt WD, et al (1998) Cardiovascular disease risk factors are lower in African-American vegans compared to lacto-ovo-vegetarians. J Am Coll Nutr 17: 425–434. 9791838 10.1080/07315724.1998.10718789

[35] Li D, Sinclair A, Mann N, Turner A, Ball M, et al (1999) The association of diet and thrombotic risk factors in healthy male vegetarians and meat-eaters. Eur J Clin Nutr 53: 612–619. 10477247 10.1038/sj.ejcn.1600817

[36] Agren JJ, Tormala ML, Nenonen MT, Hanninen OO (1995) Fatty acid composition of erythrocyte, platelet, and serum lipids in strict vegans. Lipids 30: 365–369. 7609607 10.1007/BF02536047

[37] Benatar J, Stewart RAH (2017) Plasma lipids and fatty acids in vegans compared to healthy controls. Heart, Lung and Circulation 26 S344.

[38] Bradbury KE, Crowe FL, Appleby PN, Schmidt JA, Travis RC, et al (2014) Serum concentrations of cholesterol, apolipoprotein A-I, and apolipoprotein B in a total of 1 694 meat-eaters, fish-eaters, vegetarians, and vegans. European journal of clinical nutrition 68: 178–183. 10.1038/ejcn.2013.248 24346473 PMC3916209

[39] Chiu Y-F, Hsu C-C, Chiu THT, Lee C-Y, Liu T-T, et al (2015) Cross-sectional and longitudinal comparisons of metabolic profiles between vegetarian and non-vegetarian subjects: a matched cohort study. British Journal of Nutrition 114: 1313–1320. 10.1017/S0007114515002937 26355190

[40] De Biase SG, Fernandes SF, Gianini RJ, Duarte JL (2007) Vegetarian diet and cholesterol and triglycerides levels. Arq Bras Cardiol 88: 35–39. 17364116 10.1590/s0066-782x2007000100006

[41] Elorinne A-L, Alfthan G, Erlund I, Kivimäki H, Paju A, et al (2016) Food and Nutrient Intake and Nutritional Status of Finnish Vegans and Non-Vegetarians. PLoS ONE 11: e0148235 10.1371/journal.pone.0148235 26840251 PMC4739591

[42] Famodu AA, Osilesi O, Makinde YO, Osonuga OA (1998) Blood pressure and blood lipid levels among vegetarian, semi-vegetarian, and non-vegetarian native Africans. Clin Biochem 31: 545–549. 9812174 10.1016/s0009-9120(98)00067-8

[43] Fisher M, Levine PH, Weiner B, Ockene IS, Johnson B, et al (1986) The effect of vegetarian diets on plasma lipid and platelet levels. Arch Intern Med 146: 1193–1197. 3718107

[44] Fontana L, Meyer TE, Klein S, Holloszy JO (2007) Long-term low-calorie low-protein vegan diet and endurance exercise are associated with low cardiometabolic risk. Rejuvenation Res 10: 225–234. 10.1089/rej.2006.0529 17518696

[45] Goff LM, Bell JD, So PW, Dornhorst A, Frost GS (2005) Veganism and its relationship with insulin resistance and intramyocellular lipid. Eur J Clin Nutr 59: 291–298. 10.1038/sj.ejcn.1602076 15523486

[46] Gojda J, Patkova J, Jacek M, Potockova J, Trnka J, et al (2013) Higher insulin sensitivity in vegans is not associated with higher mitochondrial density. Eur J Clin Nutr 67: 1310–1315. 10.1038/ejcn.2013.202 24149445

[47] Haddad EH, Berk LS, Kettering JD, Hubbard RW, Peters WR (1999) Dietary intake and biochemical, hematologic, and immune status of vegans compared with nonvegetarians. The American Journal of Clinical Nutrition 70: 586s–593s. 10.1093/ajcn/70.3.586s 10479236

[48] Krajcovicova-Kudlackova M, Blazicek P, Babinska K, Kopcova J, Klvanova J, et al (2000) Traditional and alternative nutrition—levels of homocysteine and lipid parameters in adults. Scand J Clin Lab Invest 60: 657–664. 11218148 10.1080/00365510050216385

[49] Kritchevsky D, Tepper SA, Goodman G (1984) Diet, nutrition intake, and metabolism in populations at high and low risk for colon cancer. Relationship of diet to serum lipids. The American Journal of Clinical Nutrition 40: 921–926. 10.1093/ajcn/40.4.921 6486100

[50] Kuchta A, Lebiedzinska A, Fijalkowski M, Galaska R, Kreft E, et al (2016) Impact of plant-based diet on lipid risk factors for atherosclerosis. Cardiol J 23: 141–148. 10.5603/CJ.a2016.0002 26779974

[51] Lin C-K, Lin D-J, Yen C-H, Chen S-C, Chen C-C, et al (2010) Comparison of Renal Function and Other Health Outcomes in Vegetarians versus Omnivores in Taiwan. Journal of Health, Population, and Nutrition 28: 470–475. 20941898 10.3329/jhpn.v28i5.6155 PMC2963769

[52] Newby PK, Tucker KL, Wolk A (2005) Risk of overweight and obesity among semivegetarian, lactovegetarian, and vegan women. Am J Clin Nutr 81: 1267–1274. 10.1093/ajcn/81.6.1267 15941875

[53] Orlich MJ, Singh PN, Sabate J, Jaceldo-Siegl K, Fan J, et al (2013) Vegetarian dietary patterns and mortality in Adventist Health Study 2. JAMA Intern Med 173: 1230–1238. 10.1001/jamainternmed.2013.6473 23836264 PMC4191896

[54] Orlov SN, Agren JJ, Hanninen OO, Nenonen MT, Lietava J, et al (1994) Univalent cation fluxes in human erythrocytes from individuals with low or normal sodium intake. J Cardiovasc Risk 1: 249–254. 7621305 10.1177/174182679400100310

[55] Pettersen BJ, Anousheh R, Fan J, Jaceldo-Siegl K, Fraser GE (2012) Vegetarian diets and blood pressure among white subjects: results from the Adventist Health Study-2 (AHS-2). Public health nutrition 15: 1909–1916. 10.1017/S1368980011003454 22230619 PMC3443300

[56] Sambol SZ, Stimac D, Orlic ZC, Guina T (2009) Haematological, biochemical and bone density parameters in vegetarians and non-vegetarians. West Indian Med J 58: 512–517. 20583676

[57] Sanders TA, Ellis FR, Dickerson JW (1978) Studies of vegans: the fatty acid composition of plasma choline phosphoglycerides, erythrocytes, adipose tissue, and breast milk, and some indicators of susceptibility to ischemic heart disease in vegans and omnivore controls. Am J Clin Nutr 31: 805–813. 10.1093/ajcn/31.5.805 645628

[58] Schupbach R, Wegmuller R, Berguerand C, Bui M, Herter-Aeberli I (2015) Micronutrient status and intake in omnivores, vegetarians and vegans in Switzerland. Eur J Nutr. 10.1007/s00394-015-1079-7 26502280

[59] Timko CA, Hormes JM, Chubski J (2012) Will the real vegetarian please stand up? An investigation of dietary restraint and eating disorder symptoms in vegetarians versus non-vegetarians. Appetite 58: 982–990. 10.1016/j.appet.2012.02.005 22343135

[60] Thomas HV, Davey GK, Key TJ (1999) Oestradiol and sex hormone-binding globulin in premenopausal and post-menopausal meat-eaters, vegetarians and vegans. British Journal of Cancer 80: 1470–1475. 10.1038/sj.bjc.6690546 10424753 PMC2363084

[61] Bradbury KE, Crowe FL, Appleby PN, Schmidt JA, Travis RC, et al (2014) Serum concentrations of cholesterol, apolipoprotein A-I and apolipoprotein B in a total of 1694 meat-eaters, fish-eaters, vegetarians and vegans. Eur J Clin Nutr 68: 178–183. 10.1038/ejcn.2013.248 24346473 PMC3916209

[62] Food and Drug Administration: 21 CFR 50. Protection of Human Subjects. Subpart 3(g). date accessed: June 22, 2011,http://www.accessdata.fda.gov/scripts/cdrh/cfdocs/cfcfr/cfrsearch.cfm?cfrpart=50&ashowfr=1.

[63] Newby P, Muller D, Hallfrisch J, Andres R, Tucker KL (2004) Food patterns measured by factor analysis and anthropometric changes in adults. The American Journal of Clinical Nutrition 80: 504–513. 10.1093/ajcn/80.2.504 15277177

[64] Pan W-H, Wu H-J, Yeh C-J, Chuang S-Y, Chang H-Y, et al (2011) Diet and Health Trends in Taiwan: Comparison of Two Nutrition and Health Surveys from 1993–1996 and 2005–2008. Asia Pacific Journal of Clinical Nutrition 20: 238–250. 21669593

[65] Kennedy ET, Bowman SA, Spence JT, Freedman M, King J (2001) Popular diets: correlation to health, nutrition, and obesity. J Am Diet Assoc 101: 411–420. 10.1016/S0002-8223(01)00108-0 11320946

[66] Ramage S, Farmer A, Eccles KA, McCargar L (2014) Healthy strategies for successful weight loss and weight maintenance: a systematic review. Appl Physiol Nutr Metab 39: 1–20. 10.1139/apnm-2013-0026 24383502

[67] Al-Khudairy L, Hartley L, Clar C, Flowers N, Hooper L, et al (2015) Omega 6 fatty acids for the primary prevention of cardiovascular disease. Cochrane Database Syst Rev: Cd011094 10.1002/14651858.CD011094.pub2 26571451

[68] Hooper L, Summerbell CD, Thompson R, Sills D, Roberts FG, et al (2012) Reduced or modified dietary fat for preventing cardiovascular disease. Cochrane Database Syst Rev 5: CD002137. 10.1002/14651858.CD002137.pub3 PMC6486029 22592684

[69] Brouwer IA, Wanders AJ, Katan MB (2010) Effect of animal and industrial trans fatty acids on HDL and LDL cholesterol levels in humans—a quantitative review. PLoS One 5: e9434 10.1371/journal.pone.0009434 20209147 PMC2830458

[70] Silverman MG, Ference BA, Im K, et al (2016) Association between lowering ldl-c and cardiovascular risk reduction among different therapeutic interventions: A systematic review and meta-analysis. JAMA 316: 1289–1297. 10.1001/jama.2016.13985 27673306

[71] Ettehad D, Emdin CA, Kiran A, Anderson SG, Callender T, et al Blood pressure lowering for prevention of cardiovascular disease and death: a systematic review and meta-analysis. The Lancet 387: 957–967. 10.1016/S0140-6736(15)01225-8 26724178

[72] Yokoyama Y, Nishimura K, Barnard ND, Takegami M, Watanabe M, et al (2014) Vegetarian diets and blood pressure: a meta-analysis. JAMA Intern Med 174: 577–587. 10.1001/jamainternmed.2013.14547 24566947

[73] Wing RR, Lang W, Wadden TA, Safford M, Knowler WC, et al (2011) Benefits of Modest Weight Loss in Improving Cardiovascular Risk Factors in Overweight and Obese Individuals With Type 2 Diabetes. Diabetes Care 34: 1481–1486. 10.2337/dc10-2415 21593294 PMC3120182

[74] Neter JE, Stam BE, Kok FJ, Grobbee DE, Geleijnse JM (2003) Influence of Weight Reduction on Blood Pressure. A Meta-Analysis of Randomized Controlled Trials 42: 878–884. 10.1161/01.HYP.0000094221.86888.AE 12975389

[75] Chiu S, Bergeron N, Williams PT, Bray GA, Sutherland B, et al (2016) Comparison of the DASH (Dietary Approaches to Stop Hypertension) diet and a higher-fat DASH diet on blood pressure and lipids and lipoproteins: a randomized controlled trial. Am J Clin Nutr 103: 341–347. 10.3945/ajcn.115.123281 26718414 PMC4733264

[76] Craig WJ (2009) Health effects of vegan diets. The American Journal of Clinical Nutrition 89: 1627S–1633S. 10.3945/ajcn.2009.26736N 19279075

[77] Barnard ND, Cohen J, Jenkins DJA, Turner-McGrievy G, Gloede L, et al (2006) A Low-Fat Vegan Diet Improves Glycemic Control and Cardiovascular Risk Factors in a Randomized Clinical Trial in Individuals With Type 2 Diabetes. Diabetes Care 29: 1777–1783. 10.2337/dc06-0606 16873779

[78] Lee Y-M, Kim S-A, Lee I-K, Kim J-G, Park K-G, et al (2016) Effect of a Brown Rice Based Vegan Diet and Conventional Diabetic Diet on Glycemic Control of Patients with Type 2 Diabetes: A 12-Week Randomized Clinical Trial. PLOS ONE 11: e0155918 10.1371/journal.pone.0155918 27253526 PMC4890770

[79] Radnitz C, Beezhold B, DiMatteo J (2015) Investigation of lifestyle choices of individuals following a vegan diet for health and ethical reasons. Appetite 90: 31–36. 10.1016/j.appet.2015.02.026 25725486

[80] Flint AJ, Rexrode KM, Hu FB, Glynn RJ, Caspard H, et al (2010) Body mass index, waist circumference, and risk of coronary heart disease: a prospective study among men and women. Obesity research & clinical practice 4: e171–e181. 21116472 10.1016/j.orcp.2010.01.001 PMC2992336

[81] Burkert NT, Freidl W, Grossschadel F, Muckenhuber J, Stronegger WJ, et al (2014) Nutrition and health: different forms of diet and their relationship with various health parameters among Austrian adults. Wien Klin Wochenschr 126: 113–118. 10.1007/s00508-013-0483-3 24343044

[82] Kristal AR, Peters U, Potter JD (2005) Is It Time to Abandon the Food Frequency Questionnaire? Cancer Epidemiology Biomarkers & Prevention 14: 2826–2828. 10.1158/1055-9965.EPI-12-ED1 16364996

[83] Navarro A, Osella AR, Guerra V, Munoz SE, Lantieri MJ, et al (2001) Reproducibility and validity of a food-frequency questionnaire in assessing dietary intakes and food habits in epidemiological cancer studies in Argentina. J Exp Clin Cancer Res 20: 365–370. 11718216

[84] Schaefer EJ, Augustin JL, Schaefer MM, Rasmussen H, Ordovas JM, et al (2000) Lack of efficacy of a food-frequency questionnaire in assessing dietary macronutrient intakes in subjects consuming diets of known composition. The American Journal of Clinical Nutrition 71: 746–751. 10.1093/ajcn/71.3.746 10702168

