# Supplementary material for: Cardiometabolic risk factors in vegans; A meta-analysis of observational studies
Source: PLoS One. 2018 Dec 20;13(12):e0209086. doi: 10.1371/journal.pone.0209086 (PMC6301673; doi:10.1371/journal.pone.0209086)

**S1 File: Supplementary document containing supplementary figures**

**Fig A:** Total energy intake (Mega joules) in vegans compared to omnivores.


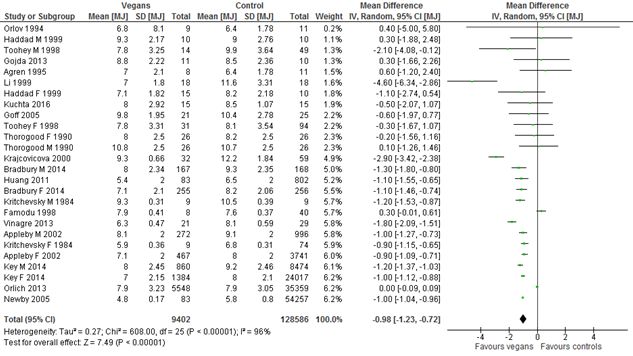


**Fig B:** Total fat intake in grams per day in vegans compared to omnivores**.**


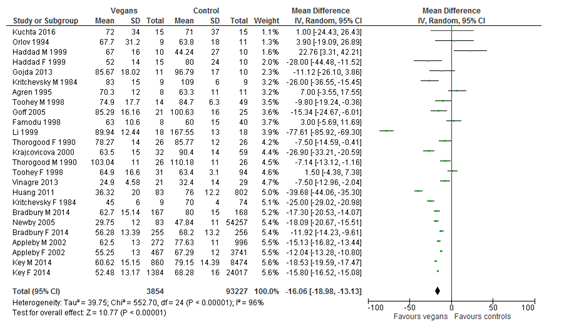


**Fig C:** Saturated fat intake in grams per day in vegans compared to omnivores.


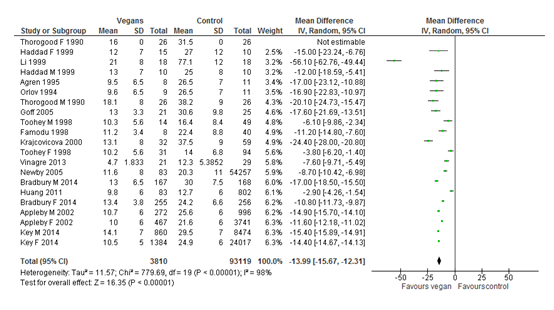


**Fig D:** Monounsaturated fat intake in grams per day in vegans compared to omnivores.


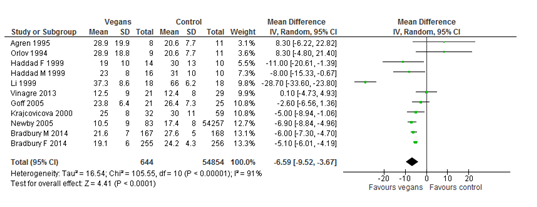


**Fig E:** Polyunsaturated fat intake in grams per day in vegans compared to omnivores.


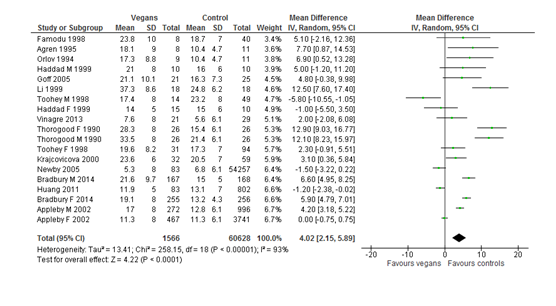


**Fig F:** Protein intake in grams per day in vegans compared to omnivores.

**
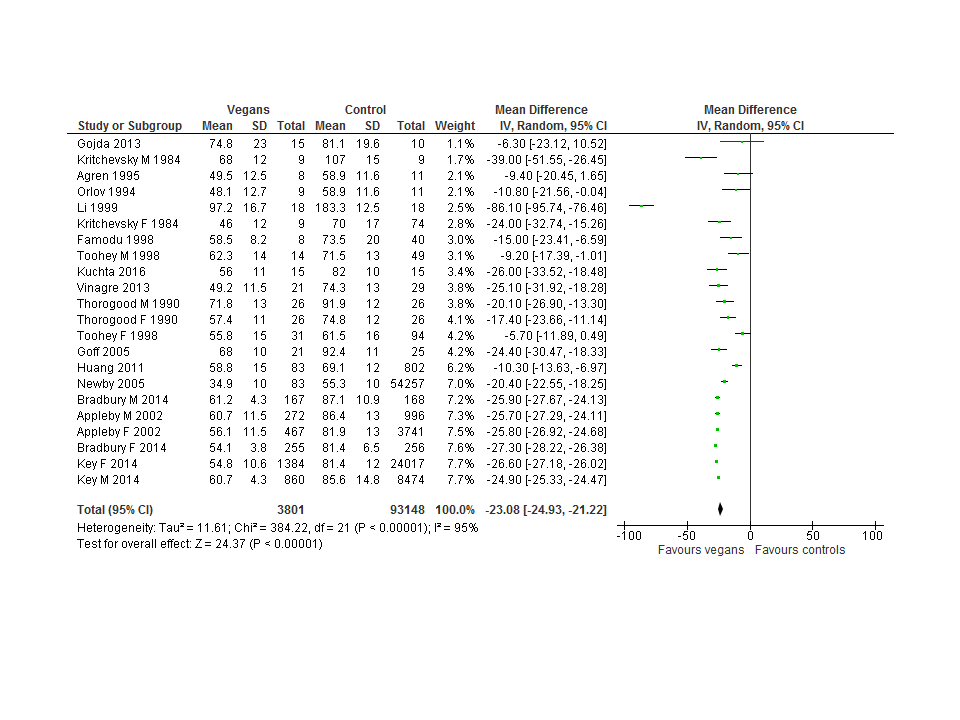
**

**Fig G:** Carbohydrate intake in grams per day in vegans compared to omnivores.

**
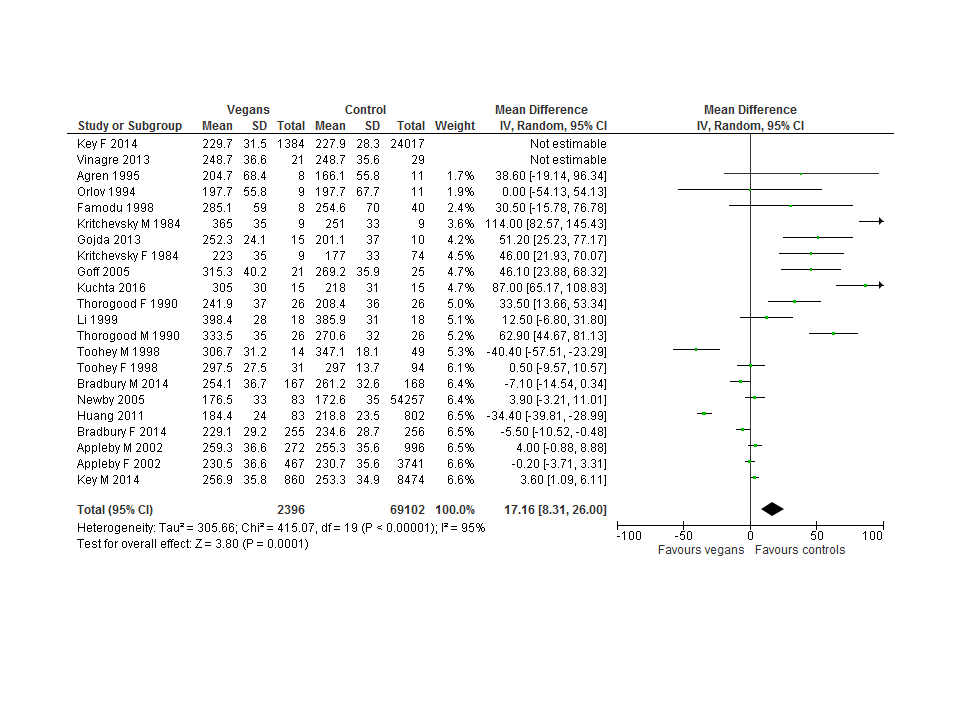
**

**Fig H:** Body mass index (kg/m2) in vegans compared to omnivores.

**
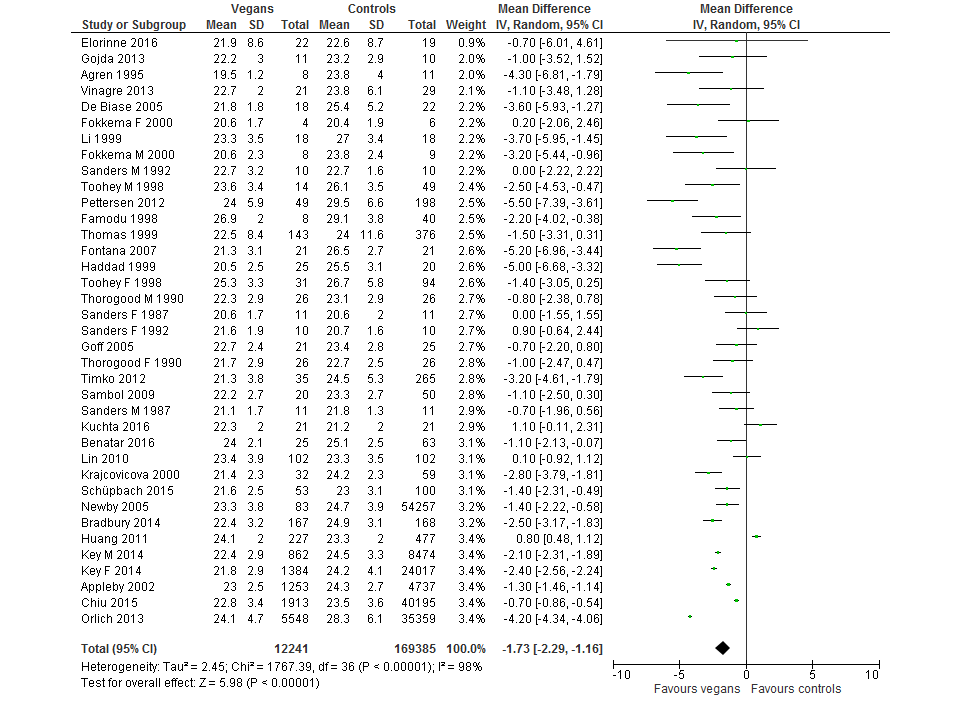
**

**Fig I:** Funnel plot of body mass index (kg/m2) in vegans compared to omnivores.

**
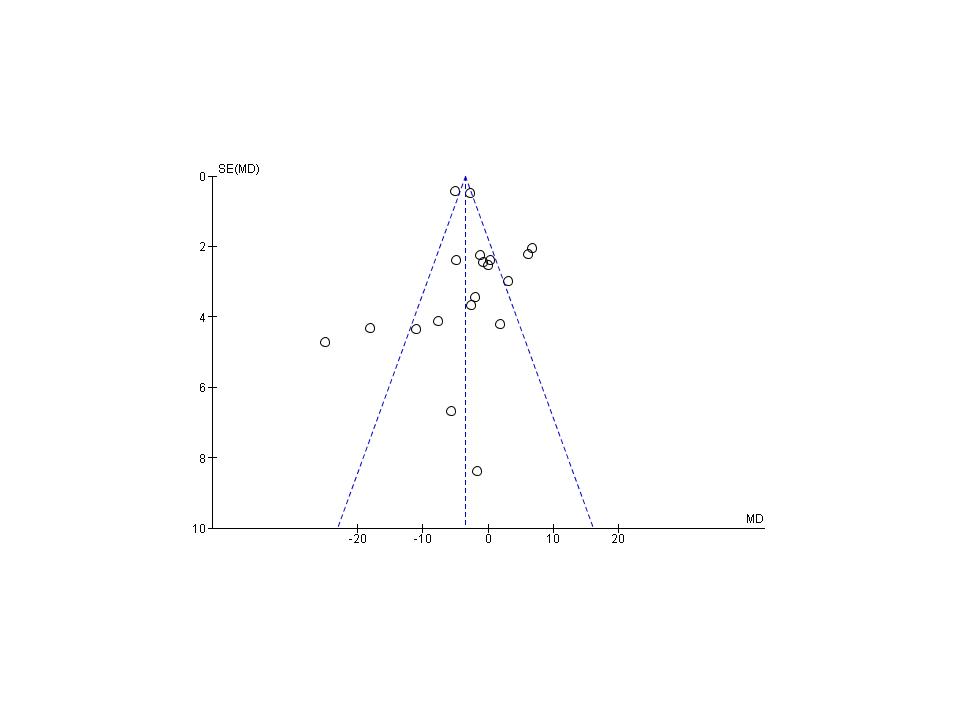
**

**Fig J:** Waist circumference (cm) in vegans compared to omnivores.


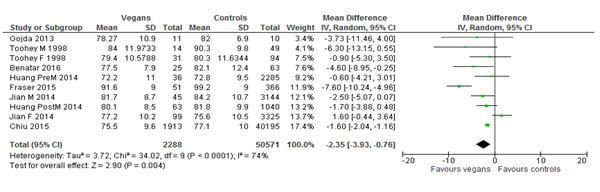


**Fig K:** Funnel plot waist circumference (cm) in vegans compared to omnivores.


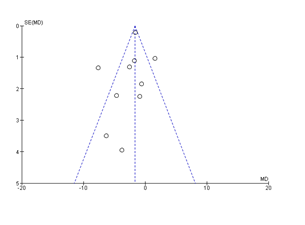


**Fig L:** Fasting blood glucose (mmol/L) in vegans compared to omnivores.


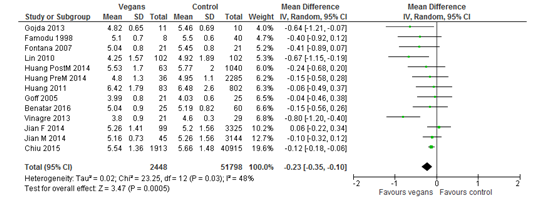


**Fig M:** Funnel plot fasting blood glucose (mmol/L) in vegans compared to omnivores.


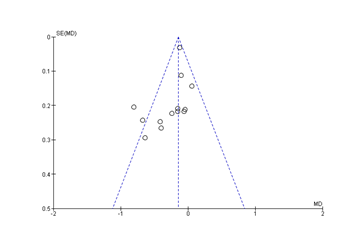


**Fig N:** Insulin resistance as calculated by the homeostasis model (insulin resistance) in vegans compared to omnivores.


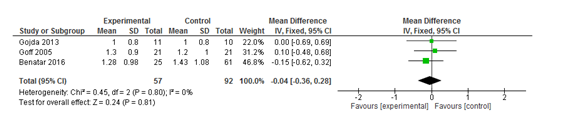


**Fig O:** Insulin resistance as calculated by the homeostasis model (insulin resistance) in vegans compared to omnivores.


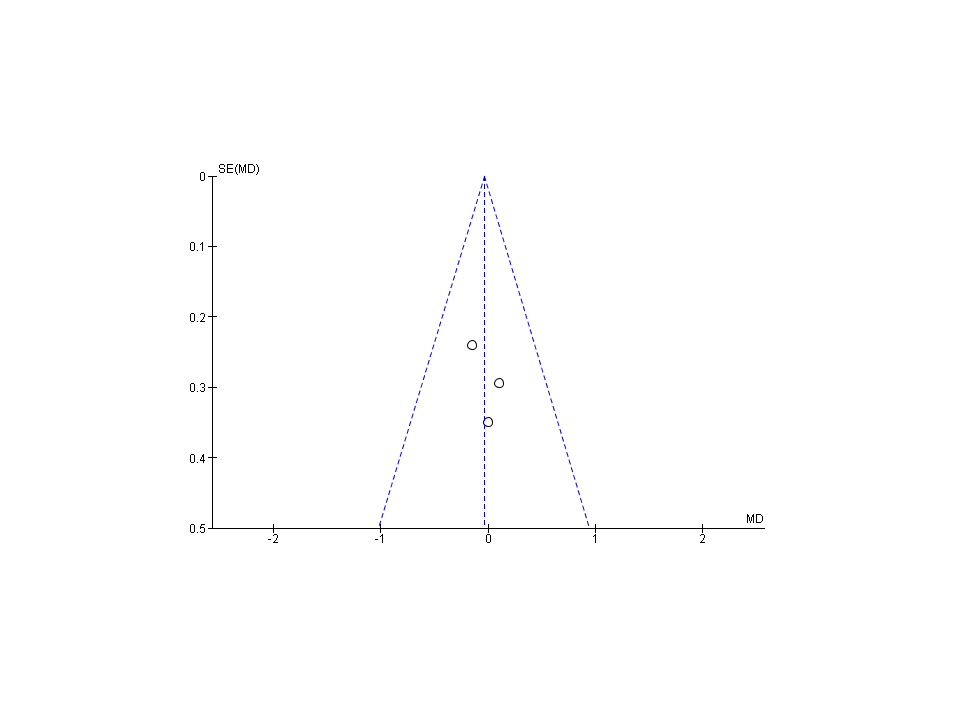


**Fig P: Low density lipoprotein cholesterol (mmol/L) level in vegans compared to omnivores.**

**
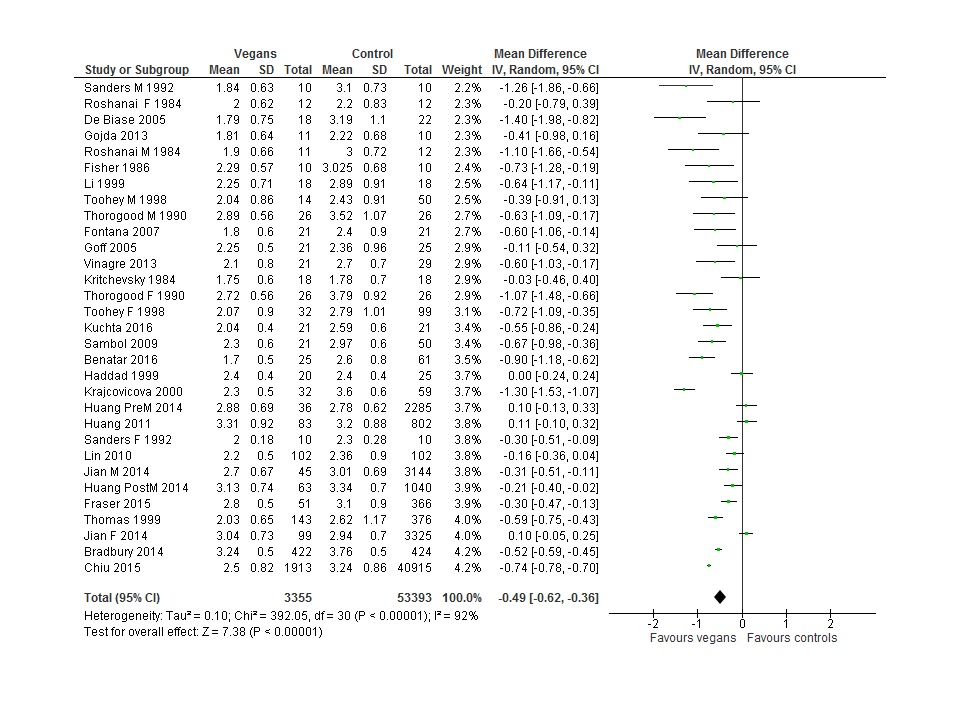
**

**Fig Q: Funnel plot low density lipoprotein cholesterol (mmol/L) level in vegans compared to omnivores**.


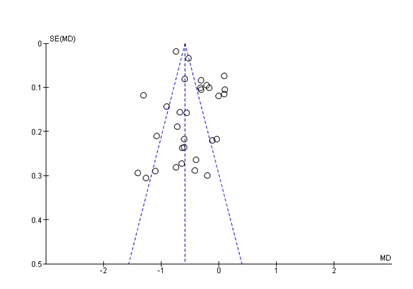


**Fig R: Triglyceride (mmol/L) levels in vegans compared to omnivores.**


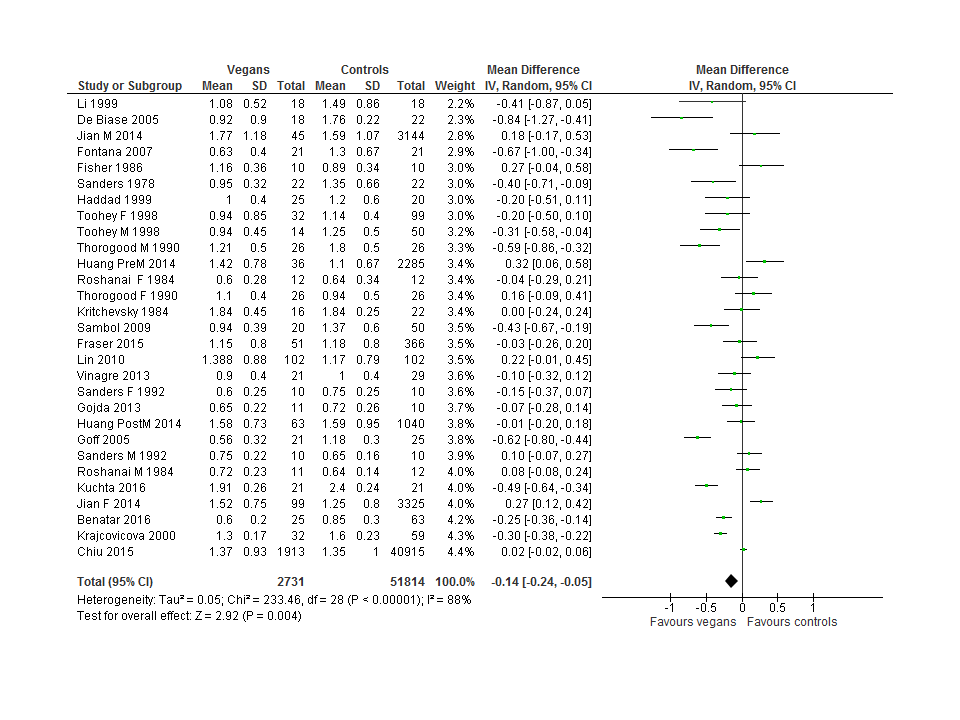


**Fig S: Funnel plot triglyceride (mmol/L) levels in vegans compared to omnivores.**


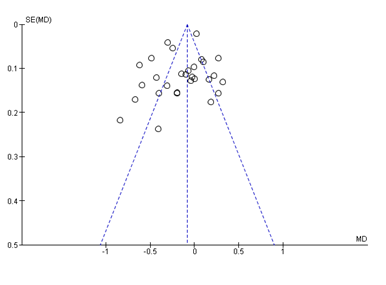


**Fig T: Systolic blood pressure (mmHg) in vegans compared to omnivores.**


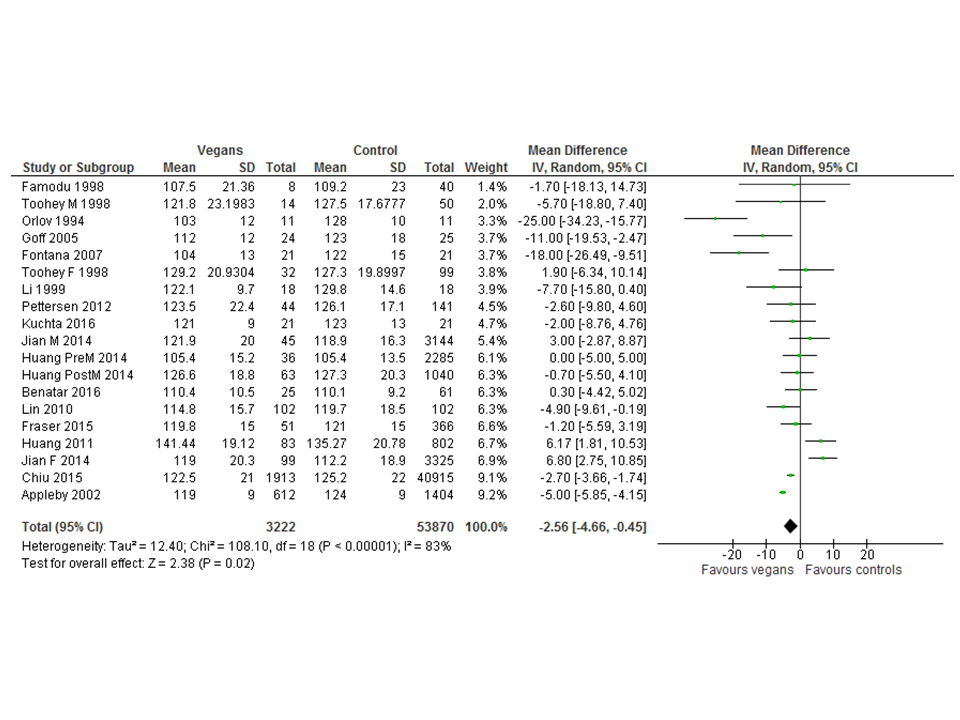


**Fig U: Funnel plot systolic blood pressure (mmHg) in vegans compared to omnivores.**


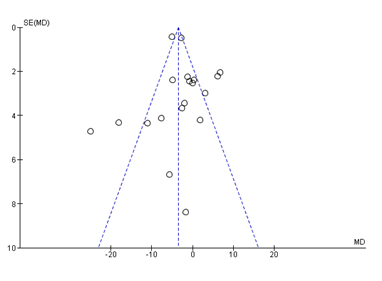


**Fig V: Diastolic blood pressure (mmHg) in vegans compared to omnivores.**


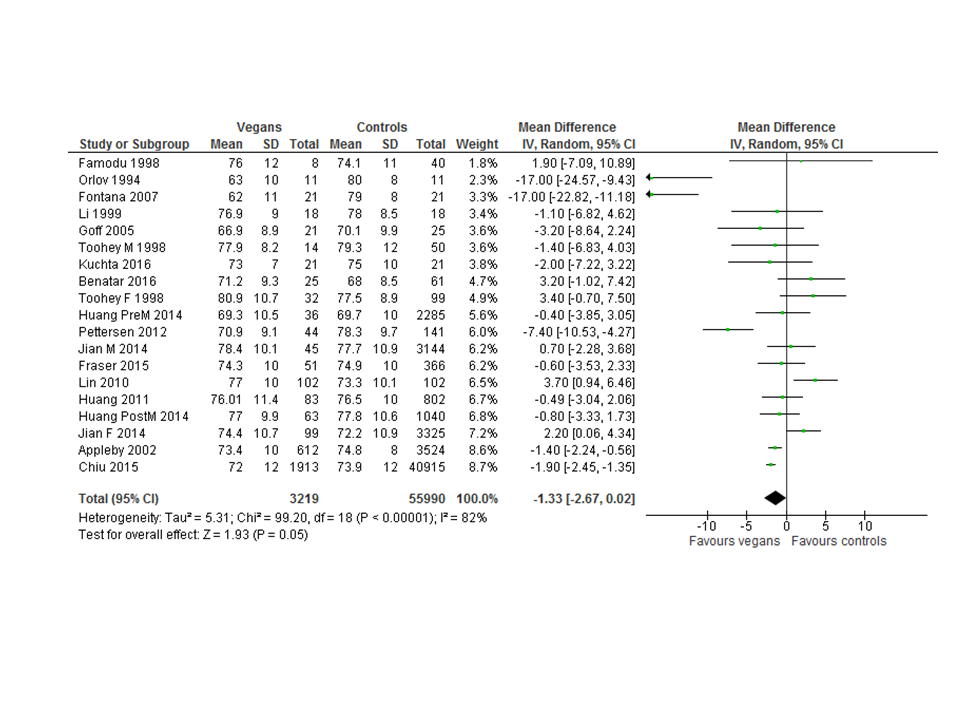


**Fig W: Funnel plot diastolic blood pressure (mmHg) in vegans compared to omnivores.**


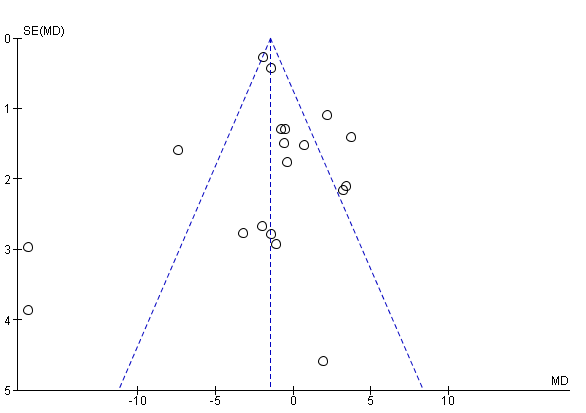

Supplement: S1 File — Supplementary document containing supplementary figures Figure A: Total energy intake (Mega joules) in vegans compared to omnivores Figure B: Total fat intake in grams per day in vegans compared to omnivores Figure C: Saturated fat intake in grams per day in vegans compared to omnivores Figure D: Monounsaturated fat intake in grams per day in vegans compared to omnivores Figure E: Polyunsaturated fat intake in grams per day in vegans compared to omnivores Figure F: Protein intake in grams per day in vegans compared to omnivores Figure G: Carbohydrate intake in grams per day in vegans compared to omnivores Figure H: Body mass index (kg/m2) in vegans compared to omnivores. Figure I: Funnel plot of body mass index (kg/m2) in vegans compared to omnivores. Figure J: Waist circumference (cm) in vegans compared to omnivores. Figure K: Funnel plot waist circumference (cm) in vegans compared to omnivores Figure L: Fasting blood glucose (mmol/L) in vegans compared to omnivores Figure M: Funnel plot fasting blood glucose (mmol/L) in vegans compared to omnivores Figure N: Insulin resistance as calculated by the homeostasis model (insulin resistance) in vegans compared to omnivores. Figure O: Insulin resistance as calculated by the homeostasis model (insulin resistance) in vegans compared to omnivores. Figure P: Low density lipoprotein cholesterol (mmol/L) level in vegans compared to omnivores. Figure Q: Funnel plot low density lipoprotein cholesterol (mmol/L) level in vegans compared to omnivores. Figure R: Triglyceride (mmol/L) levels in vegans compared to omnivores. Figure S: Funnel plot triglyceride (mmol/L) levels in vegans compared to omnivores. Figure T: Systolic blood pressure (mmHg) in vegans compared to omnivores. Figure U: Funnel plot systolic blood pressure (mmHg) in vegans compared to omnivores. Figure V: Diastolic blood pressure (mmHg) in vegans compared to omnivores. Figure W: Funnel plot diastolic blood pressure (mmHg) in vegans compared to omnivores. (DOCX) [file pone.0209086.s001.docx]
