# Supplementary material for: Cardiometabolic risk factors in vegans; A meta-analysis of observational studies
Source: PLoS One. 2018 Dec 20;13(12):e0209086. doi: 10.1371/journal.pone.0209086 (PMC6301673; doi:10.1371/journal.pone.0209086)
Supplement: S3 File — (DOCX) [file pone.0209086.s003.docx]

**Cardiometabolic risk factors in vegans –a systematic review of case control studies.**

**Study Protocol**

**Jocelyne Benatar**

**Ralph Stewart**

Dietary habits are known to be an important determinant of health. According to current guidelines, a healthy dietary pattern is high in vegetables, fruit, whole grains, seafood, legumes, and nuts, includes a modest amount of low- and non-fat dairy products. It is also low in red and processed meat, in sugar-sweetened foods and beverages, and refined grains.[[1](#_ENREF_1),[2](#_ENREF_2)] In the randomised Dietary Approach to Adults with systolic Hypertension (DASH) study, this dietary pattern was shown to reduce blood pressure,[[3](#_ENREF_3)] and insulin resistance.[[4](#_ENREF_4)] The Mediterranean diet is similar, but includes a higher intake of fruit and lower intake of dairy food. [[5](#_ENREF_5)] The Mediterranean diet has been associated with reduced cardiovascular events,[[6](#_ENREF_6),[7](#_ENREF_7)] diabetes,[[8](#_ENREF_8),[9](#_ENREF_9)] obesity, reduced blood pressure[[9](#_ENREF_9)] and modest beneficial effects on LDL cholesterol.[[10](#_ENREF_10)] The ‘healthy vegetarian eating pattern’[[1](#_ENREF_1)] has been associated with lower LDL- cholesterol[[11](#_ENREF_11),[12](#_ENREF_12)] and blood pressure.[[13](#_ENREF_13)] These diets include at least some dairy food, eggs and processed foods that contain trans fatty acids and saturated fatty acids that may affect lipid levels. [[14](#_ENREF_14)]

In contrast vegans adhere strictly to a plant based diet and are careful to avoid all animal products. The vegan diet patterns is typically low in saturated fat and has more dietary fibre and phytochemicals including phytosterols, phenolics, carotenoids, flavonoids, indoles, saponins, and sulphide [[15-18](#_ENREF_15)] that may affect LDL-cholesterol levels.[[19](#_ENREF_19)]

Evaluating cardiometabolic risk factors in vegans provides an opportunity to evaluate the effects of a strict plant based diet. A number of studies have reported cardiometabolic risk factors in vegans compared to controls, but many are small, and most evaluate only some risk factors. Studies have been undertaken in diverse geographies, and been published over many years, during which dietary patterns have changed. The hypothesis of this study is that vegans have lower blood pressure and LDL- cholesterols than omnivores. A meta-analysis of these studies allows evaluation of clinically important but modest effects on cardiometabolic risk factors, and whether these are consistent across diverse populations.

**Methods**

The review will be conducted according to Meta-analysis Of Observational Studies in Epidemiology (MOOSE) statement. [[20](#_ENREF_20)] JB will screen all abstracts and titles and JB and RS will review the full text to determine eligibility. JB will abstract data from eligible studies. Questions arising during data abstraction will be resolved by discussion. Through an iterative process, a standard list will be used to extract descriptive, methodological and key variables from all eligible studies. Data to be extracted is

- year of publication
- the primary aim of the study
- population characteristics
- funding source
- gender
- whether a food frequency questionnaire was used
- how long participants were vegan
- estimates of effect and standard deviations.

If data was not included in the published report corresponding authors will be contacted. The Newcastle Ottawa Scale (NOS) [[21](#_ENREF_21)] will assess the quality of each study.

Search Criteria

Inclusion

- cross sectional studies in healthy adults following a vegan diet longer than 6 months
- Control group who ate an omnivorous diet.
- Individual study authors defined what it was to be a vegan.
- Sufficient data to calculate estimates of effect with standard deviations on at least one of the following: body mass index, waist circumference, blood pressure, triglycerides, LDL cholesterol, fasting glucose and insulin resistance.

Exclusion

- Participants with diabetes, hypertension or vascular disease
- Participants on lipid or glucose lowering medication.
- any other intervention
- commentaries
- reviews
- not in English
- Duplicate publications from the same study.

Searches were performed of literature published from 1960 through to June 2017

Three separate types of searches will be undertaken on multiple occasions using the keywords

1. vegan AND weight
2. vegan AND blood pressure
3. vegan AND cholesterol
4. vegan AND lipids
5. vegan AND cardiometabolic
6. vegan AND insulin
7. vegan AND blood sugar
8. vegan AND insulin resistance
9. vegan AND cardiovascular

Humans studies only and publications only from 1960 onwards considered. Duplicates between the searches will be removed.

Statistical analysis

The inverse-variance method will be used to pool mean differences to yield an overall effect size with 95% confidence intervals. Where standard deviations or confidence intervals are not available despite contacting authors, the mean SD of all other studies is used.

Total energy (kilojoules), carbohydrate, total, saturated, polyunsaturated and mono unsaturated fat and protein intake (grams/day) will be collected and the mathematical weighted mean will be calculated.

Heterogeneity will be assessed by a Chi square test and I2 statistic. A fixed effects model will be used when heterogeneity is not present (I2=0) and a random effects model when statistical heterogeneity (I2≥1%) is present. The meta-analysis will also repeated using a fixed effects model to assess the effects of small studies on results.[[22](#_ENREF_22)] A p-value of <0.05 is considered statistically significant. Studies are presented in Forrest plots in order of statistical power.

Subgroups and Sensitivity analysis

Sensitivity analysis will be undertaken to assess effects of studies that deviate significantly from the standard error of the total study result or studies where baseline values differ significantly from the mean baseline. Subgroup analysis will be conducted for size of study (<50 or >50 vegan participants), geography (North America, Europe, Asia and other), and for date of publication (< 2000, 2000-2010, >2010). If possible this will also be done for gender. Funnel plots are used to evaluate for possible publication bias.[[23](#_ENREF_23)].

The Statistical analyses will be performed using RevMan software version 5·2 (The Nordic Cochrane Centre, The Cochrane Collaboration, Copenhagen). Subgroup analysis followed guidelines suggested by Wang. [[24](#_ENREF_24)]

**References**

1. (2015) 2015-2020 Dietary Guidelines for Americans. United States of America: USDA Center for Nutrition Policy and Promotion.

2. Perk J, De Backer G, Gohlke H, Graham I, Reiner Z, et al. (2012) European Guidelines on cardiovascular disease prevention in clinical practice (version 2012). The Fifth Joint Task Force of the European Society of Cardiology and Other Societies on Cardiovascular Disease Prevention in Clinical Practice (constituted by representatives of nine societies and by invited experts). Developed with the special contribution of the European Association for Cardiovascular Prevention & Rehabilitation (EACPR). Eur Heart J 33: 1635-1701.

3. Appel LJ, Moore TJ, Obarzanek E, Vollmer WM, Svetkey LP, et al. (1997) A clinical trial of the effects of dietary patterns on blood pressure. DASH Collaborative Research Group. N Engl J Med 336: 1117-1124.

4. Liese AD, Nichols M, Sun X, D'Agostino RB, Jr., Haffner SM (2009) Adherence to the DASH Diet is inversely associated with incidence of type 2 diabetes: the insulin resistance atherosclerosis study. Diabetes Care 32: 1434-1436.

5. Estruch R, Ros E, Salas-Salvadó J, Covas MI, Corella D, et al. (2013) Primary prevention of cardiovascular disease with a Mediterranean diet. N Engl J Med 368.

6. Estruch R, Ros E, Salas-Salvado J, Covas MI, Pharm D, et al. (2013) Primary Prevention of Cardiovascular Disease with a Mediterranean Diet. N Engl J Med: 1279-1290.

7. Kris-Etherton P, Eckel RH, Howard BV, St. Jeor S, Bazzarre TL, et al. (2001) Lyon Diet Heart Study: Benefits of a Mediterranean-Style, National Cholesterol Education Program/American Heart Association Step I Dietary Pattern on Cardiovascular Disease. Circulation 103: 1823-1825.

8. Esposito K, Giugliano D (2014) Mediterranean diet and type 2 diabetes. Diabetes Metab Res Rev 30 Suppl 1: 34-40.

9. Pérez-Martínez P, García-Ríos A, Delgado-Lista J, Pérez-Jiménez F, López-Miranda J (2011) Mediterranean diet rich in olive oil and obesity, metabolic syndrome and diabetes mellitus. Curr Pharm Des 17.

10. Rosenthal RL (2000) Effectiveness of altering serum cholesterol levels without drugs. Proceedings (Baylor University Medical Center) 13: 351-355.

11. Mann N, Pirotta Y, O'Connell S, Li D, Kelly F, et al. (2006) Fatty acid composition of habitual omnivore and vegetarian diets. Lipids 41: 637-646.

12. Appleby PN, Davey GK, Key TJ (2002) Hypertension and blood pressure among meat eaters, fish eaters, vegetarians and vegans in EPIC–Oxford. Public Health Nutrition 5: 645-654.

13. Yokoyama Y, Nishimura K, Barnard ND, et al. (2014) Vegetarian diets and blood pressure: A meta-analysis. JAMA Internal Medicine 174: 577-587.

14. Hammad S, Pu S, Jones PJ (2016) Current Evidence Supporting the Link Between Dietary Fatty Acids and Cardiovascular Disease. Lipids 51: 507-517.

15. Kritchevsky D, Tepper SA, Goodman G (1984) Diet, nutrition intake, and metabolism in populations at high and low risk for colon cancer. Relationship of diet to serum lipids. The American Journal of Clinical Nutrition 40: 921-926.

16. Huang CJ, Fan YC, Liu JF, Tsai PS (2011) Characteristics and nutrient intake of Taiwanese elderly vegetarians: evidence from a national survey. Br J Nutr 106: 451-460.

17. Goff LM, Bell JD, So PW, Dornhorst A, Frost GS (2004) Veganism and its relationship with insulin resistance and intramyocellular lipid. Eur J Clin Nutr 59: 291-298.

18. Vinagre JC, Vinagre CG, Pozzi FS, Slywitch E, Maranhao RC (2013) Metabolism of triglyceride-rich lipoproteins and transfer of lipids to high-density lipoproteins (HDL) in vegan and omnivore subjects. Nutr Metab Cardiovasc Dis 23: 61-67.

19. Ras RT, Geleijnse JM, Trautwein EA (2014) LDL-cholesterol-lowering effect of plant sterols and stanols across different dose ranges: a meta-analysis of randomised controlled studies. The British Journal of Nutrition 112: 214-219.

20. Stroup DF, Berlin JA, Morton SC, Olkin I, Williamson GD, et al. (2000) Meta-analysis of observational studies in epidemiology: a proposal for reporting. Meta-analysis Of Observational Studies in Epidemiology (MOOSE) group. Jama 283: 2008-2012.

21. Wells GA SB, O’Connell D, Peterson J, Welch V, Losos M, et al. The Newcastle-Ottawa Scale (NOS) for assessing the quality if nonrandomized studies in meta-analyses.

22. Tufanaru C, Munn Z, Stephenson M, Aromataris E (2015) Fixed or random effects meta-analysis? Common methodological issues in systematic reviews of effectiveness. Int J Evid Based Healthc 13: 196-207.

23. Egger M, Davey Smith G, Schneider M, Minder C (1997) Bias in meta-analysis detected by a simple, graphical test. Bmj 315: 629-634.

24. Wang R, Lagakos SW, Ware JH, Hunter DJ, Drazen JM (2007) Statistics in Medicine — Reporting of Subgroup Analyses in Clinical Trials. New England Journal of Medicine 357: 2189-2194.
